# Estimated global and regional economic burden of genital herpes simplex virus infection among 15–49 year-olds in 2016

**DOI:** 10.1186/s44263-024-00053-6

**Published:** 2024-07-02

**Authors:** Nathorn Chaiyakunapruk, Shaun Wen Huey Lee, Puttarin Kulchaitanaroaj, Ajaree Rayanakorn, Haeseon Lee, Katharine Jane Looker, Raymond Hutubessy, Sami L. Gottlieb

**Affiliations:** 1 Department of Pharmacotherapy, College of Pharmacy, University of Utah, Salt Lake City, UT 84112 USA; 2 School of Pharmacy, Monash University Malaysia, Jalan Lagoon Selatan, Subang Jaya, Selangor, Malaysia; 3 School of Pharmacy, Taylor’s University, Jalan Taylors, Subang Jaya, Selangor, Malaysia; 4 Department of Pharmacy Practice and Science, College of Pharmacy, University of Iowa, Iowa City, IA USA; 5 Mathematical and Economic Modelling, Mahidol-Oxford Tropical Medicine Research Unit, Faculty of Tropical Medicine, Mahidol University, Bangkok, Thailand; 6 Department of Pharmacology, Faculty of Medicine, Chiang Mai University, Chiang Mai, 50200 Thailand; 7 Population Health Sciences, Bristol Medical School, University of Bristol, Bristol, UK; 8 Department of Immunization, Vaccines and Biologicals (IVB), World Health Organization, Geneva, Switzerland; 9 Department of Sexual and Reproductive Health and Research, World Health Organization, Geneva, Switzerland

**Keywords:** HSV, Herpes simplex virus, Global estimate, Economic burden, Vaccine

## Abstract

**Background:**

Globally, herpes simplex virus (HSV)-2 and -1 infections contribute to a large disease burden, but their full economic consequences remain unclear. This study aims to estimate the global economic impact of genital HSV-2 and HSV-1 infection and its consequences for people with genital ulcer disease, neonatal herpes, and human immunodeficiency virus (HIV) infection attributable to HSV-2.

**Methods:**

Using a societal perspective, the economic burden was calculated at the country level and presented by World Health Organization (WHO) regions and World-Bank income levels. The disease burden was obtained from previously published global disease burden studies in 2016 and disaggregated for 194 countries. Estimates of healthcare resource utilisation were sourced from a literature review, and online interviews were conducted with 20 experts from all 6 WHO regions. Relevant costs were obtained from the literature and estimated in 2016 international dollars (I$).

**Results:**

Both genital HSV-2 (I$31·2 billion) and HSV-1 (I$4·0 billion) infections and their consequences were estimated to cost I$35·3 billion globally in 2016. The major economic burden was from the Americas and Western Pacific regions combined, accounting for almost two-thirds of the global burden (I$20·8 billion). High- and upper-middle-income countries bore a large proportion of the economic burden (76·6% or I$27·0 billion). Costs were driven by the large number of HSV-2 recurrences; however, even assuming conservatively that people with symptomatic herpes have on average only one episode a year, global costs were estimated at I$16·5 billion.

**Conclusions:**

The global costs of genital HSV infection and its consequences are substantial. HSV prevention interventions have the potential to avert a large economic burden in addition to disease burden; thus, efforts to accelerate HSV vaccine development are crucial.

**Supplementary Information:**

The online version contains supplementary material available at 10.1186/s44263-024-00053-6.

## Background

In 2016, approximately 13·2% of the world’s population aged 15–49 years were living with HSV-2, while 67% of the world’s population aged 0–49 years were infected with HSV-1 [1]. HSV-2 causes genital herpes, one of the most prevalent sexually transmitted infections worldwide [1, 2], characterised by recurrent, self-limiting outbreaks of painful genital lesions collectively termed genital ulcer disease (GUD) [1]. Moreover, HSV-2 infection can almost triple the risk of sexually acquired HIV infection [3, 4]. HSV-1 is mainly transmitted by oral-oral contact to cause oral herpes, but it can also cause genital herpes [1]. Both genital HSV-1 and HSV-2 can cause neonatal infection [5] with a high fatality rate despite its rareness [6].

Concerns about HSV infection include not only its far-reaching health effects but also its impact on quality of life [7]. Genital herpes can lead to stigmatisation and detrimental effects on sexual relationships, given its lifelong and recurrent nature. Currently available HSV interventions, such as antiviral drugs, can reduce symptoms but cannot cure or prevent transmission on a population level; the development of a safe and efficacious vaccine for HSV is an important goal to reduce HSV infection and avert health and economic burden.

An assessment of the economic burden of HSV is an important step toward supporting the public health value assessment for HSV vaccine development. Nevertheless, there is poor understanding of the global economic impact of HSV infection. Previous research has mostly focussed on the United States (US) and has shown that the economic burden of HSV-2 infection was substantial and projected to rise to US $2·5 billion in 2015 [8, 9]. A more recent model among 90 low- and middle-income countries (LMICs) predicted that genital herpes could contribute to approximately US $29 billion costs in 2019, including effects on absenteeism and productivity [10]. This study aims to estimate the global economic burden of genital herpes caused by HSV-2 and HSV-1 infections in adults, pregnant women, neonatal herpes, and HIV infection attributable to HSV-2 across all six WHO regions in 2016.

## Methods

### Overview

We estimated the global economic burden based on published estimates of HSV disease outcomes for the year 2016 [1, 3, 5]. We used a societal perspective incorporating costs related to direct medical care, transportation, and productivity loss in the main analysis and a healthcare perspective including only direct medical costs in the sensitivity analysis. An ingredient-based approach, calculated by multiplying healthcare resource utilisation (HCRU) with unit costs and disease burden, was mainly used for the economic burden of all HSV-related infections, while the economic burden of HIV attributable to HSV-2 infection used the published average annual total health spending for HIV [11]. Economic burden of HSV infection including HSV-2- and HSV-1-related genital infections and HSV-2 attributable HIV infection in adults aged 15–49 years and pregnant women, prevention of neonatal herpes among pregnant women with HSV, and neonatal herpes was estimated for a 1-year period globally.

### Disease burden estimates

We disaggregated global and regional disease burden of GUD [1], neonatal herpes [5], and HSV-attributable HIV infection [3] into estimates for each of the 194 countries in all 6 WHO regions based on the proportion of country burden among regional estimates for HSV-2 infection [12]. The estimated country-specific disease burdens were age and sex stratified and available separately for HSV-2 and HSV-1 infections. Further details are in Additional file 1: Appendix 1.

### Healthcare resource utilisation

We investigated HCRU patterns among people with HSV from a previously conducted systematic review from inception to August 2020 [13] and grey literature through Google using the same keywords [13]. The results showed that most HCRU data were from the USA and other high-income countries. Therefore, we obtained the HCRU data consistently through virtual interviews of 20 experts from 12 countries representing high-, middle-, and low-income countries across all WHO regions including Australia, Brazil, China, India, Lebanon, Moldova, South Africa, Sri Lanka, the Netherlands, Uganda, the UK, and the USA.

Regarding GUD, HCRU was estimated separately for first and recurrent episodes among adults/adolescents and for pregnant women. HCRU estimates incorporated patients’ care-seeking behaviour pattern, laboratory/diagnostic tests, counselling, and treatment patterns. In addition, for pregnant women, we estimated the additional caesarean section (CS) burden attributable to HSV by considering baseline CS rates for each country [14] due to various intrapartum reasons to undergo CS.

HCRU estimates for neonatal herpes were based on three major presentations at central nervous system, disseminated organs, and the skin, eye, and/or mouth (SEM) [6, 15]. For central nervous system and disseminated diseases, different proportions would be managed as neonatal sepsis, bacterial meningitis/pneumonia, and neonatal herpes, whereas for SEM, different proportions would be managed as superficial bacterial infection, neonatal sepsis, and neonatal herpes. Further detail about HCRU and the healthcare processes can be found in Additional file 1: Appendix 2.

### Unit costs

Unit costs included direct medical costs, direct nonmedical costs, and indirect costs. All unit costs were country-specific. The unit costs of outpatient visits at a healthcare facility and inpatient visits per day were based on the estimates by WHO-CHOICE [WHO-CHOosing Interventions that are Cost Effective] [16], while the unit costs of neonatal intensive care unit and laboratory/diagnostic tests were based on literature searches from PubMed and other databases. The unit cost of counselling was constructed by applying a remuneration for a nurse providing counselling for 15 min [17, 18], and the unit cost of a pharmacist visit was computed from a 5-min pharmacist remuneration [19]. Unit costs of medication treatment were based on WHO/HAI [WHO/Health Action International project on medicine prices and availability] [20] and national-agency/ministry websites for medication costs listed on the WHO website [21]. Some unit costs, such as total healthcare costs for HIV treatment and prevention per capita, and additional costs of caesarean delivery due to HSV infection compared with vaginal delivery, were taken as a lump sum from published articles [11]. Transportation cost was based on a round trip of a local ticket from a publicly available website, namely Numbeo [22], while the indirect cost was estimated based on average wages obtained from International Labour Organization [23]. Any missing data were imputed by using the average from all other countries at the same income level and in the same region or subregion. If there were no cost data for a particular income level, a cost ratio of a similar product was used to estimate the missing values.

All costs were converted to I$ in the year 2016 to aid in the interpretation of the economic burden. The currency conversion process followed the guideline by Turner et al. [24] See Additional file 1: Appendix 3 for details.

### Economic burden estimation

The economic burden was calculated by the multiplication of country-based, HSV-type-specific disease burden of GUD among adults and pregnant women, neonatal herpes, and HSV-associated HIV infection with HCRU and associated costs for all 194 countries. The estimated economic burdens of individual countries were summed and stratified by income level for each WHO region [25, 26].

### Sensitivity analyses

We performed a series of sensitivity analyses. First, we estimated the burden using a healthcare perspective. Second, we conducted a probabilistic sensitivity analysis to determine the robustness of our estimates. The uncertainty was modelled by assigning a normal distribution to disease burden; beta, triangular, or normal distributions to HCRU; and gamma distribution to cost parameters. We adopted either 95% confidence interval or plausible ranges for HCRU and assumed ±20% to generate upper and lower limits of cost estimates. In each simulation, the disease burden, cost, and HCRU parameters were iterated for each country and summed to calculate regional and global estimates. Simulation runs consisted of 1000 iterations. We also estimated economic burden in a conservative scenario where the number of recurrent episodes was limited to one per individual. Finally, an alternative scenario was modelled, assuming that all individuals with HSV sought care and were treated based upon treatment guidelines recommended by WHO. Results were presented in terms of the 95% credible interval (CrI) which was the 2.5th and 97.5th percentile of the iterated estimates. All calculations were done using Microsoft Excel (Redmond, WA, USA). This study was reported in accordance with the Guidelines for Accurate and Transparent Health Estimates Reporting recommendations [27].

## Results

### Overall economic burden

**Fig. 1:**
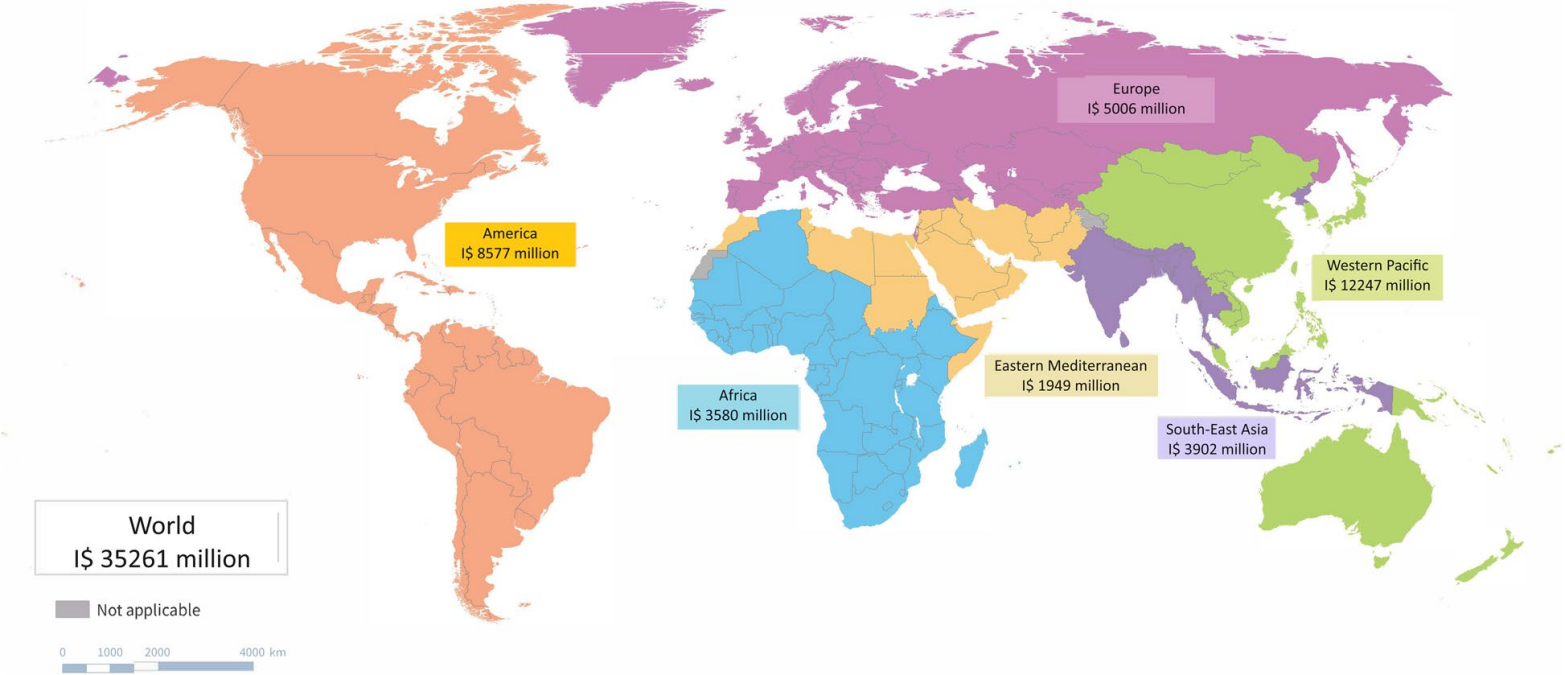
Economic burden due to HSV globally, by WHO region in 2016

Globally, the estimated economic burden of genital HSV infection and its consequences in 2016 were I$35·3 billion (Table 1 and Fig. 1). Of this, I$31·2 billion (88%) was associated with HSV-2 and I$4·0 billion with HSV-1. By WHO region, genital HSV was responsible for I$3·6 billion in the African region, I$8·6 billion in the Americas region, I$1·9 billion in the Eastern Mediterranean region, I$5·0 billion in the Europe region, I$3·9 billion in South-East Asia region, and I$12·2 billion in the Western Pacific region.

**Table 1:** Economic burden of HSV by regions

**WHO region**	**All countries**	**High-income countries**	**Upper-middle income countries**	**Low-middle income countries**	**Low-income countries**
	**GUD due to HSV-2 (I$ million)**				
**Global**	30,807	9589	13,586	6612	1020
African	3354	0.7	1367	1182	804
Americas	6486	4160	2221	91	14
Eastern Mediterranean	1749	131	735	734	149
Europe	4040	2297	1498	235	10
South-East Asia^b^	3879	-	438	3398	43
Western Pacific^c^	11,299	3001	7327	971	-
	**GUD due to HSV-1 (I$ million)**				
**Global**	3988	2219	1501	238	30
African^a^	-	-	-	-	-
Americas	1956	1443	488	21	4
Eastern Mediterranean	194	32	65	74	23
Europe	902	599	254	45	4
South-East Asia^b^	16	-	0.8	15	0.2
Western Pacific^c^	920	145	693	83	-
	**Neonatal herpes due to a maternal HSV-2 infection (I$ million)**				
**Global**	53	30	15	6	2
African	6	0.0	2	3	2
Americas	22	18	4	0.2	0.0
Eastern Mediterranean	3	1.4	0.8	1.1	0.1
Europe	10	8	2	0.2	0.0
South-East Asia^b^	1.1	-	0.1	1.0	0.0
Western Pacific^c^	10	3	6	0.6	-
	**Neonatal herpes due to a maternal HSV-1 infection (I$ million)**				
**Global**	61	41	18	2	0.1
African	0.0	0.0	0.0	0.0	0.0
Americas	36	28	8	0.3	0.0
Eastern Mediterranean	1.5	0.5	0.4	0.5	0.1
Europe	13	9	3	0.3	0.0
South-East Asia^b^	0.1	-	0.0	0.1	0.0
Western Pacific^c^	11	3	7	0.7	-
	**HIV attributable to HSV-2 (I$ million)**				
**Global**	352	92	199	47	14
African	219	0.0	168	38	14
Americas	78	56	21	0.2	0.2
Eastern Mediterranean	0.8	0.2	0.3	0.3	0.0
Europe	41	34	4	3	0.0
South-East Asia^b^	6	-	0.5	5	0.0
Western Pacific^c^	8	2	5	0.9	-

Totals may vary due to rounding. The numbers are given to the nearest integer from model estimates

^a^No estimates were generated for the African region for HSV-1 due to lack of information on disease burden

^b^No countries within the South-East Asia region were classified as high income

^c^No countries within the Western Pacific region were classified as low income

The proportion of economic burden due to HSV infections differed by country income level. The economic burden in both high- and upper-middle-income countries contributed to more than three quarters of the global HSV burden in 2016 (I$27·0 billion, 76·6%). By country income levels, HSV was responsible for I$11·9 billion in high-income countries, I$15·1 billion in upper-middle-income countries, I$6·9 billion in lower-middle-income countries, and I$1·1 billion in low-income countries. The largest economic burden was due to HSV-2 in all countries irrespective of income level. In comparison, genital HSV-1 was responsible for I$2·3 billion in high-income countries, I$1·5 billion in upper-middle-income countries, I$0·2 billion in lower-middle-income countries, and I$0·03 billion in low-income countries.

By type of costs, direct medical costs were responsible for the majority (I$22·0 billion; 62·3%) of the total costs associated with HSV infection, followed by indirect costs (I$12·3 billion; 34·9%) and direct nonmedical costs (I$1·0 billion; 2·8%). The direct cost of HSV was generally higher in the Americas and Western Pacific regions compared to the African and South-East Asia regions (Table 1 and Additional file 1: Table S8).

### Disease-specific burden

#### GUD due to genital herpes among adults and genital herpes in pregnancy

In all countries, HSV-2 GUD was responsible for I$30·8 billion (88·5%) in economic burden compared to I$4·0 billion due to HSV-1 GUD. By WHO region, GUD due to HSV-2 was responsible for the following: I$3·4 billion in the African region, I$6·5 billion in the Americas region, I$1·7 billion in the Eastern Mediterranean region, I$4·0 billion in the Europe region, I$3·9 billion in the South-East Asia region, and I$11·3 billion in the Western Pacific region. Conversely, GUD due to HSV-1 was responsible for I$2·0 billion in the Americas region, I$0·2 billion in Eastern Mediterranean region, I$0·9 billion in Europe region, I$0·02 billion in South-East Asia region, and I$0·9 billion in Western Pacific region. Stratified by country income levels, the largest economic burden due to HSV-2 was observed in upper-middle-income countries, followed by high-income countries except for the America and Europe. Genital HSV-1 was responsible for the largest economic burden in high-income countries followed by upper-middle-income countries (Table 2).

**Table 2:** Cost comparison of economic burden of HSV by WHO region and country income group (2016)

	**GUD due to HSV-1 (I$ million, %)**	**GUD due to HSV-2 (I$ million, %)**	**Neonatal herpes due to HSV-1 (I$ million, %)**	**Neonatal herpes due to HSV-2 (I$ million, %)**	**HIV-attributable to HSV-2 (I$ million, %)**
**By WHO region**					
**Global**	3988 (100%)	30,807 (100%)	61 (100%)	53 (100%)	352 (100%)
African	-	3354 (10·9%)	0.0 (0·0%)	6 (12·1%)	219 (62·3%)
Americas	1956 (49·0%)	6486 (21·1%)	36 (58·9%)	22 (41·1%)	78 (22·2%)
Eastern Mediterranean	194 (4·9%)	1749 (5·7%)	1·5 (2·4%)	3 (6·5%)	0·8 (0·2%)
Europe	902 (22·6%)	4040 (13·1%)	13 (20·6%)	10 (18·9%)	41 (11·5%)
South-East Asia	16 (0·4%)	3879 (12·6%)	0·1 (0·2%)	1·1 (2·0%)	6 (1·6%)
Western Pacific	920 (23·1%)	11,299 (36·7%)	11 (17·4%)	10 (19·4%)	8 (2·2%)
**By World Bank income level**					
**Global**	3988 (100%)	30,807 (100%)	61 (100%)	53 (100%)	352 (100%)
High	2219 (55·6%)	9589 (31·1%)	41 (66·9%)	30 (56·7%)	92 (26·1%)
Upper-middle	1501 (37·6%)	13,586 (44·1%)	18 (29·7%)	15 (28·7%)	199 (56·5%)
Lower-middle	238 (6·0%)	6612 (21·5%)	2 (3·3%)	6 (11·3%)	47 (13·4%)
Low	30 (0·8%)	1020 (3·3%)	0·1 (0·2%)	2 (3·3%)	14 (4·0%)

*WHO *World Health Organization, *HIV *Human immunodeficiency virus

#### Neonatal herpes

In all countries, the estimated economic burden of neonatal herpes was I$53·4 million due to HSV-2 and I$60·6 million due to HSV-1. The primary contributors were the Americas, solely responsible for almost half of the worldwide economic burden for both HSV-1 and HSV-2 neonatal herpes.

#### HIV attributable to HSV-2

Across all countries, the estimated economic burden of HIV attributable to HSV-2 was I$352·0 million. The largest burden was in the African region, which accounted for I$219·4 million in cost, mainly due to the direct medical costs associated with antiretroviral therapy for 1 year.

### Sensitivity analyses

Using a healthcare perspective, the global economic burden of GUD due to HSV was I$22·0 billion. Of this, HSV-2 GUD was responsible for I$19·0 billion, neonatal herpes due to HSV-2 was responsible for I$44 million, and HIV attributable to HSV-2 was responsible for I$292 million (see Additional file 1: Table S8). To evaluate the potential variability of economic burden, assuming an idealistic scenario where all patients received treatment based upon guidelines, the economic burden of HSV was estimated to increase to I$80·3 billion. Importantly, the cost would be more than double in the African region if all patients sought and received treatment and care. In the scenario limiting the number of recurrent episodes of GUD to one per individual, the estimated global economic burden due to HSV-2 accounted for I$12.5 billion, while the combined economic burden of genital HSV infection, encompassing both HSV-1 and HSV-2 in 2016, was estimated at I$16.5 billion.

The probabilistic sensitivity analyses showed that economic burden of genital HSV infection and its consequences was I$35·7 (95% *CrI*, I$22·6–I$50·8) billion in 2016. Genital, neonatal, and HIV associated with HSV-2 accounted for $31.5 billion (95% *CrI*, I$20·3–I$43·4 billion), while genital and neonatal HSV-1 covered I$4·2 (95% *CrI*, I$2·3–I$7·4) billion. It was observed that the estimates were highly affected by the disease burden and costs of HSV treatment. The full details are in Additional file 1: Table S9.

## Discussion

This study provides the first global estimates of the economic burden of genital HSV infection and its consequences. Based on data from 194 countries, we comprehensively estimated that, in 2016, HSV cost the world economy I$35·3 billion in healthcare expenditure and productivity losses. The economic burden of HSV was unequally distributed across regions and disproportionately affected high- and upper-middle-income countries. Geographically, the highest economic burden was observed in the Americas and Western Pacific regions, which collectively contributed nearly two-thirds of the global economic burden at I$20·8 billion. This is likely due to higher proportions of individuals seeking care, higher utilisation of diagnostic tests, and higher therapy costs, compared to other regions.

The global economic burden was greatest for HSV-2 GUD, 7.7-fold higher compared to HSV-1 GUD (I$30·8 billion vs I$4·0 billion). This is not surprising as the global burden of HSV-2 GUD is much higher, given more frequent sexual transmission and higher recurrences rates for HSV-2. Moreover, while neonatal herpes and HIV attributable to HSV-2 were estimated to cost I$466 million, both have a very high cost of treatment per case compared to HSV GUD.

The magnitude of economic burden did not necessarily correspond to the size of disease burden. For example, while the number of people with HSV GUD was the highest in the African region (59 million) [1], the number of individuals estimated to be seeking and receiving care was relatively smaller compared to other regions. Sensitivity analyses assuming that all individuals with symptomatic genital HSV infection would be treated according to treatment guidelines suggest that the burden would more than double, mainly due to an increase in economic burden from the African and European regions. Economic burden of neonatal herpes was higher for HSV-1 compared to HSV-2 (I$60·6 million vs I$53·4 million). The disease burden of neonatal herpes due to HSV-1 was highest in the Americas region, while the African region had a much higher disease burden of neonatal herpes due to HSV-2 [5]. However, the economic burden of neonatal herpes was higher in the Americas region because there was assumed to be more HCRU and higher costs in the Americas region compared with the African region.

Most of the economic burden was contributed by direct medical cost. Given the variation in access and HCRU pattern across countries, it is possible to project that the direct medical cost associated with HSV infection would be higher when patients have more access and receive recommended care, especially in LMICs [28]. Given that some of parameters were based on experts’ opinions about HCRU per GUD episode, any overestimation of HCRU would be magnified with multiple GUD recurrences per person. Thus, we also performed an analysis using a conservative approach of limiting the number of recurrent episodes to one per individual. This endeavour is meant to circumvent the challenges associated with variability in reported values used in the model and aims to provide a lower bound of economic burden under a conservative scenario.

The results highlight the importance of a concerted effort in accelerating the development of HSV vaccines. Provided that HSV vaccines could reduce the number of genital HSV infections in the population or reduce the frequency or severity of HSV GUD outbreaks, the substantial economic costs could be averted. Additionally, as people with HSV-2 infection are more vulnerable to contracting HIV infection [29], a vaccine against HSV may also be useful in reducing HIV incidence. This is particularly important in regions where the prevalence of HIV infection is high, such as the African region, where we estimated that HIV attributable to HSV-2 infection contributed around 10% of the total economic burden of HSV in the region.

Even though there are no prior global economic burden estimates, it is crucial to compare our estimates with other previous estimates in the literature. When compared to a recent study by Silva et al. [10] that evaluated the economic losses due to genital herpes in 90 LMICs, the total economic burden due to HSV-2, derived by limiting our analysis to the 90 countries in that study, was I$20 billion, which was lower than the Silva’s finding of $29 billion. Variations in data sources regarding care-seeking behaviours and absenteeism, along with differing treatment practices due to study periods, might contribute to these differences in estimated costs. Nevertheless, this consistency will be important evidence to support validity of the global economic burden estimates.

Several limitations should be pointed out. First, we did not include any costs associated with nongenital HSV outcomes, including oral HSV-1 infection, HSV keratitis, and herpes encephalitis. Second, we applied a cross-sectional 1-year period of 2016, due to the availability and completeness of information for all relevant genital HSV-related outcomes. Importantly, our study did not consider the lifetime disease burden and treatment costs as well as changes in treatment-seeking behaviour, which could be substantial. Third, our estimates were limited by the scarcity of HCRU data for some regions such as Africa and South-East Asia. Fourth, the economic burden estimate of GUD was only for people aged 15–49 years old. Extending our estimates to include burden in older populations would mean making more assumptions in many key input variables. Fifth, the analysis was based only on the estimated treatment utilisation. If assuming that all patients were treated based upon current recommended treatment guidelines, the economic burden of HSV would have increased more than double. Finally, there was limited information on the disease burden and healthcare-seeking behaviours of individuals with HSV in each of the 194 countries. We had to disaggregate regional burden of disease to the country level by making assumptions around the contribution of individual countries as well as to rely on the HCRU patterns described in literature [13] and the expert interviews. As such, future estimates of the economic burden of HSV could be further refined with the granular data on the HCRU and additional related adverse events. It is important to note that our study, based on 2016 data, may not fully capture the current landscape. Future research incorporating any updated data, including on changes in awareness of and HCRU for HSV, are warranted. Nevertheless, our analysis is the first and most comprehensive estimates of global economic burden of HSV infection with best available data.

## Conclusions

HSV infection is a global health issue that results in substantial economic losses. Our global economic burden estimates for HSV in 2016 provide compelling evidence on the importance of investing in development of HSV prevention and control interventions. Such interventions have the potential not only to improve outcomes of affected populations worldwide but also to avert a large economic burden attributable to HSV.

## Supplementary Information

**Additional file 1:** **Appendix 1.** Estimation of disease burden. **Appendix 2.** Healthcare resource utilization. **Fig. S1.** Overview of healthcare resource utilization for HSV related to GUD in adults and adolescents. **Fig. S2.** Overview of healthcare resource utilization for HSV related to GUD in pregnancy. **Fig. S3.** Overview of healthcare resource utilization related to typical presentations of neonatal herpes. **Fig. S4.** Overview of neonatal herpes clinical presentations. **Table S1.** Summary of healthcare resource utilization estimates for HSV GUD in adults and adolescents, by region based upon expert opinion. **Table S2.** Summary of healthcare resource utilization estimates for HSV related to GUD in pregnancy, by region based upon expert opinion. **Table S3.** Summary of healthcare resource utilization estimates for HSV related to typical presentations of neonatal herpes, by region based upon expert opinion. **Appendix 3.** Unit costs. **Table S4.** Types of unit costs in the analysis. Appendix 4: Healthcare spending attributable to disease. **Table S5.** Estimates of economic burden associated with HSV-2 calculated by matching 90 low- and middle-income countries reported in existing literature. **Table S6.** Breakdown of cost associated with HSV-2. **Table S7.** Breakdown of cost associated with HSV-1. **Table S8.** Annual burden of cost related to HSV in 2016. **Table S9.** Distributional economic impact of HSV from probabilistic analyses. **Table S10.** Distributional economic impact (in millions I$) of HSV assuming idealistic practice where treatment guidelines were adhered to.

## Data Availability

All data are included in the main manuscript and its supporting files.

## Abbreviations

CS: Caesarean section
CrI: Credible interval
GUD: Genital ulcer disease
HCRU: Healthcare resource utilisation
HSV: Herpes simplex virus
HIV: Human immunodeficiency virus
I$: International dollars
LMICs: Low- and middle-income countries
SEM: Skin, eye, and/or mouth
WHO: World Health Organization
