# Supplementary material for: Estimated global and regional economic burden of genital herpes simplex virus infection among 15–49 year-olds in 2016
Source: BMC Glob Public Health. 2024 Jul 2;2:42. doi: 10.1186/s44263-024-00053-6 (PMC11618196; doi:10.1186/s44263-024-00053-6)
Supplement: Supplementary file 1 — Additional file 1: Appendix 1. Estimation of disease burden. Appendix 2. Healthcare resource utilization. Fig. S1. Overview of healthcare resource utilization for HSV related to GUD in adults and adolescents. Fig. S2. Overview of healthcare resource utilization for HSV related to GUD in pregnancy. Fig. S3. Overview of healthcare resource utilization related to typical presentations of neonatal herpes. Fig. S4. Overview of neonatal herpes clinical presentations. Table S1. Summary of healthcare resource utilization estimates for HSV GUD in adults and adolescents, by region based upon expert opinion. Table S2. Summary of healthcare resource utilization estimates for HSV related to GUD in pregnancy, by region based upon expert opinion. Table S3. Summary of healthcare resource utilization estimates for HSV related to typical presentations of neonatal herpes, by region based upon expert opinion. Appendix 3. Unit costs. Table S4. Types of unit costs in the analysis. Appendix 4: Healthcare spending attributable to disease. Table S5. Estimates of economic burden associated with HSV-2 calculated by matching 90 low- and middle-income countries reported in existing literature. Table S6. Breakdown of cost associated with HSV-2. Table S7. Breakdown of cost associated with HSV-1. Table S8. Annual burden of cost related to HSV in 2016. Table S9. Distributional economic impact of HSV from probabilistic analyses. Table S10. Distributional economic impact (in millions I$) of HSV assuming idealistic practice where treatment guidelines were adhered to. [file 44263_2024_53_MOESM1_ESM.docx]

**Estimated Global and Regional Economic Burden of Genital Herpes Simplex Virus Infection among 15-49 year-olds in 2016**

Nathorn Chaiyakunapruk^1,2^, Shaun Wen Huey Lee^2,3^, Puttarin Kulchaitanaroaj^4,5^

Ajaree Rayanakorn^6^, Haeseon Lee^1^, Katharine Jane Looker^7^, Raymond Hutubessy^8^, Sami L. Gottlieb^9^

^1^ Department of Pharmacotherapy, College of Pharmacy, University of Utah, Salt Lake City, Utah, USA

^2^ School of Pharmacy, Monash University Malaysia, Jalan Lagoon Selatan, Selangor, Malaysia

^3^ School of Pharmacy, Taylor’s University, Jalan Taylors, Selangor, Malaysia

^4^ Department of Pharmacy Practice and Science, College of Pharmacy, University of

Iowa, Iowa City, Iowa, USA

^5^ Mathematical and Economic Modelling, Mahidol-Oxford Tropical Medicine Research Unit, Faculty of Tropical Medicine, Mahidol University, Bangkok, Thailand

^6^ Department of Pharmacology, Faculty of Medicine, Chiang Mai University, Chiang Mai, Thailand

^7^ Population Health Sciences, Bristol Medical School, University of Bristol, Bristol, UK

^8^ Department of Immunization, Vaccines and Biologicals (IVB), World Health Organization, Geneva, Switzerland

^9^ Department of Sexual and Reproductive Health and Research, World Health Organization, Geneva, Switzerland

**Corresponding author**:

Nathorn Chaiyakunapruk

Department of Pharmacotherapy

University of Utah College of Pharmacy

30 South 2000 East, Room 4964

Salt Lake City, UT 84112

Office: 801.585.3092

[nathorn.chaiyakunapruk@utah.edu](mailto:nathorn.chaiyakunapruk@utah.edu)

**Additional File 1**

**Table of Contents**

[Appendix 1: Estimation of disease burden 3](#_Toc159254667)

[1.1 Population data 3](#_Toc159254668)

[1.2 HSV-related GUD burden 3](#_Toc159254669)

[1.3 HIV burden attributable to HSV 3](#_Toc159254670)

[1.4 Pregnancy and neonatal herpes due to HSV 3](#_Toc159254671)

[Appendix 2: Healthcare resource utilization 5](#_Toc159254672)

[2.1 Adults/adolescents and pregnancy 5](#_Toc159254673)

[2.2 Neonatal herpes 10](#_Toc159254674)

[Appendix 3: Unit costs 18](#_Toc159254675)

[3.1 Unit cost of outpatient visits at a healthcare facility 19](#_Toc159254676)

[3.2 Unit cost of outpatient visits at a pharmacy 19](#_Toc159254677)

[3.3 Unit cost of inpatient visits 19](#_Toc159254678)

[3.4 Unit costs of laboratory or diagnostic tests 20](#_Toc159254679)

[3.5 Unit cost of counselling 20](#_Toc159254680)

[3.6 Unit costs of treatment and suppressive therapy 20](#_Toc159254681)

[3.7 Unit cost of transportation 20](#_Toc159254682)

[3.8 Wages 20](#_Toc159254683)

[3.9 Spending for new cases of HIV/AIDS attributable to HSV infection 20](#_Toc159254684)

[3.10 Unit costs of disease management on neonates 21](#_Toc159254685)

[3.11 Unit costs of vaginal delivery and caesarean delivery 21](#_Toc159254686)

[Appendix 4: Healthcare spending attributable to disease 23](#_Toc159254687)

[4.1 Comparison of estimates of economic burden associated with HSV with existing literature 23](#_Toc159254688)

[References 28](#_Toc159254689)

# **Appendix 1: Estimation of disease burden**

## **Population data**

A comprehensive set of population data stratified by age groups and sexes was obtained from the 2016 UN Population Prospectus from the UN Department of Economic and Social Affairs, Population Division, Population Estimates and Projections Section for all the 194 countries used in the current estimates.

The data compiled by UNAIDS for their HIV/AIDS estimation process was used as our main source of data for producing estimates of HIV burden attributable to HSV. As several countries had no updated national-level data for HIV/AIDS estimates, we calculated the regional average of all needed inputs and used them as a proxy for these data.

## **1.2 HSV-related GUD burden**

We used the most recently available published estimates on global and regional cases of HSV GUD for 2016, by Looker and colleagues, as the basis to calculate the disease burden of HSV.^1^ As the estimates were reported based on WHO regions, we requested data from the Institute for Health Metrics and Evaluation (IHME), who provided the 2017 Global Burden of Diseases prevalence estimates of HSV infection at the country level.^2^ Using the data from IHME, we subsequently grouped countries into the six WHO regions, and calculated the ratio of infection burden based upon the share of population in each region. We then multiplied the ratio for each country with the estimates of GUD burden for each region. We subsequently divided the country-specific estimates of individuals with HSV GUD into people with first or recurrent GUD episodes by subtypes (either HSV-1 or HSV-2), assuming that these were consistent with the previous estimates (the ratio of the number of people with a first episode and the number of people with recurrences by region) from Looker et al.^1^ To assess the economic burden, it is essential to consider the total number of repeated GUD episodes in recurrent patients. We utilized the episode estimates provided in the Looker's study^1^ guided by two principles: (1) each individual with GUD is associated with at least one episode, and (2) an individual with a first episode of GUD is associated with the only one episode. When these principles were applied, we observed that in people with recurrent GUD due to HSV-2, the number of recurrent episodes was approximately five (5.4 for Africa, 5.3 for America, 4.9 for Eastern Mediterranean, and 5.2 for Europe, 6.0 for South East Asia, 5.3 for Western Pacific, and totally 5.4 for global).

## **1.3 HIV burden attributable to HSV**

We used the same approach described above for GUD burden estimation to generate individual country HIV burden estimates attributable to HSV based on HIV incidence data^3^ obtained from the official UNAIDS estimates.^4^

## **1.4 Pregnancy and neonatal herpes due to HSV**

The same approach as previously described was used to generate the burden of pregnancy related HSV. The latest WHO global and regional estimates of HSV-1 and HSV-2 prevalence and incidence in women, which were done for 2016 and published in 2020 was used in the current estimate. We applied live birth rates by maternal age group for each individual country^5^ to determine estimates of the prevalence and incidence of maternal HSV infection during pregnancy. We then estimated the incremental number of procedures including caesarean section and HSV suppressive therapy using a simple process from the literature and our own survey among key experts across 6 regions. The dataset was from an updated data on the trends and projections of caesarean section commissioned by the WHO, from which we extracted the caesarean section rates from 154 countries (herein referred to as baseline caesarean section rates).^6^ In the event that this information was unavailable, we imputed the regional estimates for the country. We then estimated the attributable caesarean rates associated with HSV by subtracting baseline caesarean rates with the total estimated caesarean rates from the survey among key experts as described above. In the event that these rates were lower than the baseline caesarean rates, we adjusted these values and assumed that there was an increase of 3% from baseline values based upon a study by Stankiewicz Karita.^7^

To generate disaggregated estimates for neonatal herpes due to HSV by country^8^, we obtained the number of live birth rates by age group from the United Nations Population Division.^5^ A similar approach as described above was used in the current estimates, supplemented with a survey from KOLs to validate our findings.

# **Appendix 2: Healthcare resource utilization**

To supplement findings from our systematic review of HSV-related healthcare resource utilization (HCRU), we conducted virtual interviews with 20 experts from 12 countries (Australia, Brazil, China, India, Lebanon, South Africa, Sri Lanka, the Netherlands, Moldova, Uganda, the UK, and the USA) in all six WHO regions (the Americas, Africa, the Eastern Mediterranean, Europe, Southeast Asia, and the Western Pacific) to collect HCRU patterns associated with the management and care of genital HSV infection and neonatal herpes. All participants were from relevant specialties or expertise involved in the care of HSV patients including dermatology, infectious diseases, neonatology, academic, gynaecology/obstetrics, sexually transmitted infection, and public health specialists. Given that not all experts treated all aspects of HSV related infection, these experts only shared their experiences on the relevant sections as described below. Briefly, the interview comprised of 3 distinct sections.

The first section queried participants on the use of healthcare services related to patients with genital herpes symptoms in adults and adolescents (Fig. S1). The second section was related to genital herpes and neonatal herpes prevention in pregnancy (Fig. S2) while the third section was related to healthcare services related to typical presentations of neonatal herpes (Fig. S3 and S4). Data was then compiled to generate separate assumption estimates for each different WHO region, stratified by country incomes to either high-income countries (HICs) or low- and middle-income countries (LMICs) whenever possible. Separate estimation for people living with HIV was not made explicitly. Nevertheless, assumptions for the whole population, which included immunocompromised populations, were estimated.

The base-case estimate was based on the midpoint value of each range of healthcare service utilization whereas the upper and lower limit or ±25% from the mean value (for parameters without ranges) were used for sensitivity analyses. Triangular distribution for parameters with ranges was assumed. Country-specific estimates were used if they were available. Otherwise, expert assumption estimates were applied based on countries of the same income level within the same region. Estimates from other region for the same income level were applied in case estimates within the same region were not available. These extrapolations are summarized in the sections below.

## **2.1 Adults/adolescents and pregnancy**

Resource utilization estimates include proportion seeking care, laboratory/diagnostic tests, counselling, medication treatment and regimen patterns captured in each section of the survey.

Genital ulcer disease (GUD) was defined as symptoms/signs of genital herpes, such as genital blisters, ulcers, pustules, or other lesions. The aetiology of the symptoms may not be known at the time patients present for medical care. The first genital herpes symptoms/GUD noted by the patient are considered the first episode and any subsequent genital herpes symptoms/GUD are considered recurrent episodes. For the first episode, people either seek care at a healthcare facility or a pharmacy. At a healthcare facility, clinical/syndromic management and different diagnostic tests can be performed. The patients then receive treatment with HSV antivirals and/or syphilis treatment based on the practice at each setting. For those seeking care at a pharmacy, they may be prescribed or given HSV antivirals. As for recurrent episodes, patients can seek care either at a healthcare facility or a pharmacy similar to the first episode. In addition, people may have medications (HSV antivirals from previous prescriptions) at home to take for recurrences, and a proportion of patients with recurrent genital herpes would also receive suppressive antiviral treatment at some point in their life. The pattern of HCRU would vary by countries and regions (Fig. S1).

A proportion of pregnant women with HSV GUD (either first episode or recurrence episode) would seek care at a healthcare facility in addition to antenatal visits in which syndromic management and diagnostic tests can be performed. A proportion of pregnant women with known recurrent genital herpes symptoms or with a symptomatic first episode of HSV GUD would typically be given suppressive antiviral treatment from 36 weeks of gestation until delivery to prevent neonatal herpes. A proportion of pregnant women with symptomatic first episode of HSV GUD in the third trimester or suspected genital herpes lesions at the time of delivery would undergo a caesarean section to prevent neonatal herpes. The use of healthcare services would be based on the practice in each setting (Fig. S2).

The proportion of pregnant women with lesions at the time of delivery is approximately 14% among pregnant women with recurrent episodes based on the literature^9 10^ whereby one third of pregnant women infected with HSV are assumed to present with a symptomatic first episode of HSV GUD in the third trimester based on the findings from a previous published study. ^11^

HCRU for adults/adolescents with GUD and pregnancy was obtained from experts according to the methodology described above. However, values for WHO Eastern Mediterranean Region (EMR) HICs and Southeast Asian Region (SEAR) HICs were extrapolated based on estimates from the Western Pacific Region (WPR) HICs, while the WHO European Region (EUR) HICs were assumed to be similar to the Americas (AMR) HICs, respectively, due to inability of experts to provide estimates. SEAR LMIC estimates for pregnancy HCRU were used for the WHO African Region (AFR) LMICs. Given that the estimated proportion of adults/adolescents with GUD that would seek care in EUR HICs was initially 10-35% which was markedly lower than that estimated in EUR LMICs, the WHO Region of the Americas (AMR) HIC estimate of 50% was applied as it seemed more likely to be the case. AMR LMIC estimates for pregnancy HCRU and the proportion of pregnant women receiving C-section among those with suspected genital lesions at the time of delivery and those with symptomatic first episode of HSV GUD in the third trimester were applied for EUR LMICs and EMR LMICs, respectively. EUR HIC estimates were used for AMR HICs for HCRU in pregnant women with HSV GUD. Estimates from EUR HICs for the proportion of pregnant women with symptomatic first episode receiving C-section were also applied for SEAR and WPR HICs, whereas the SEAR LMIC estimate was assumed for the proportion of pregnant women receiving C-section among those with suspected lesions at the time of delivery as the EUR HIC estimate for this proportion was higher than SEAR LMICs, which was unlikely to be the case. All HCRU parameters estimated in the model for adults/adolescents and pregnant women are listed in Tables S1 and S2 respectively.

**
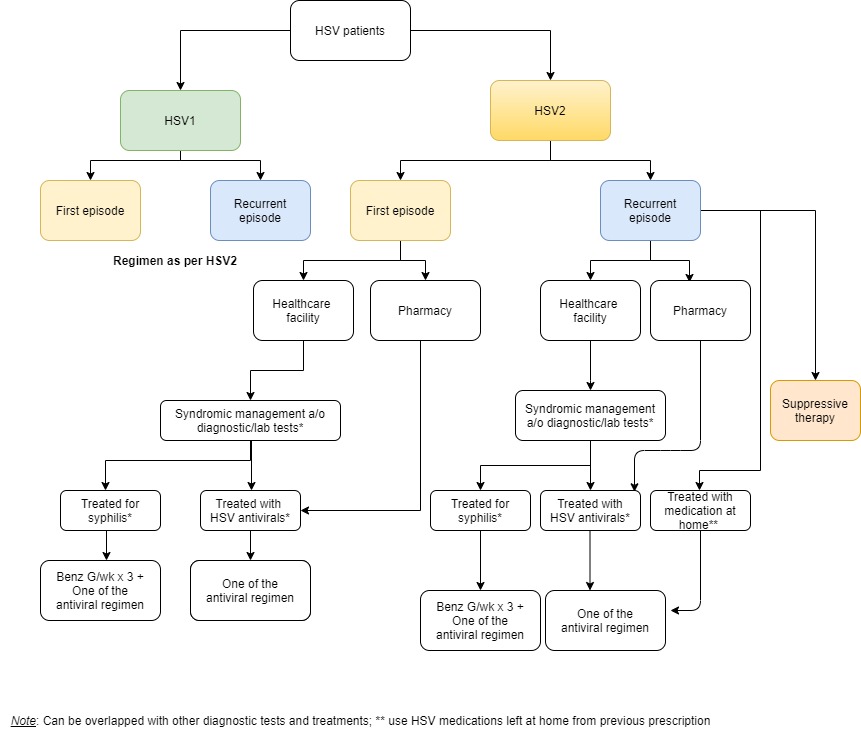
**

**Fig. S1** Overview of healthcare resource utilization for HSV related to GUD in adults and adolescents.

*Note:* The healthcare resource utilization (HCRU) nodes above represent options for which experts were asked to estimate proportions; there is also the option that people do not seek care nor get treated; *Can overlap with other diagnostic tests and treatments; **Use HSV medications at home from a previous prescription


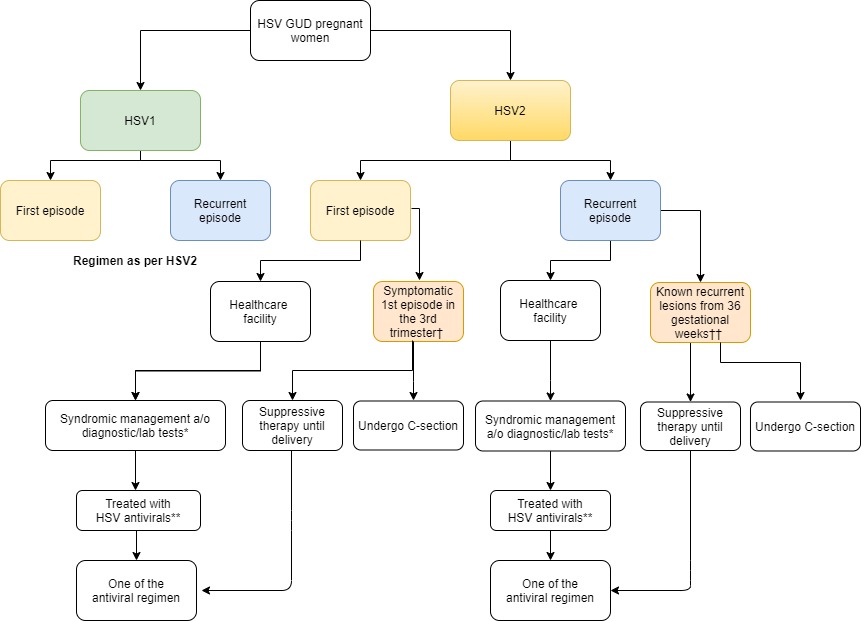


**Fig. S2** Overview of healthcare resource utilization for HSV related to GUD in pregnancy.

*Note*: The healthcare resource utilization (HCRU) nodes above represent options for which experts were asked to estimate proportions; there is also the option that people do not seek care nor get treated; *Can overlap with other diagnostic tests and treatments; ** HSV antiviral treatment pattern is similar to non-pregnant adults/adolescents with HSV GUD**;** †Pregnant women with symptomatic 1^st^ episode of GUD in the 3^rd^ trimester; †† Pregnant women with known recurrent genital herpes lesions

**
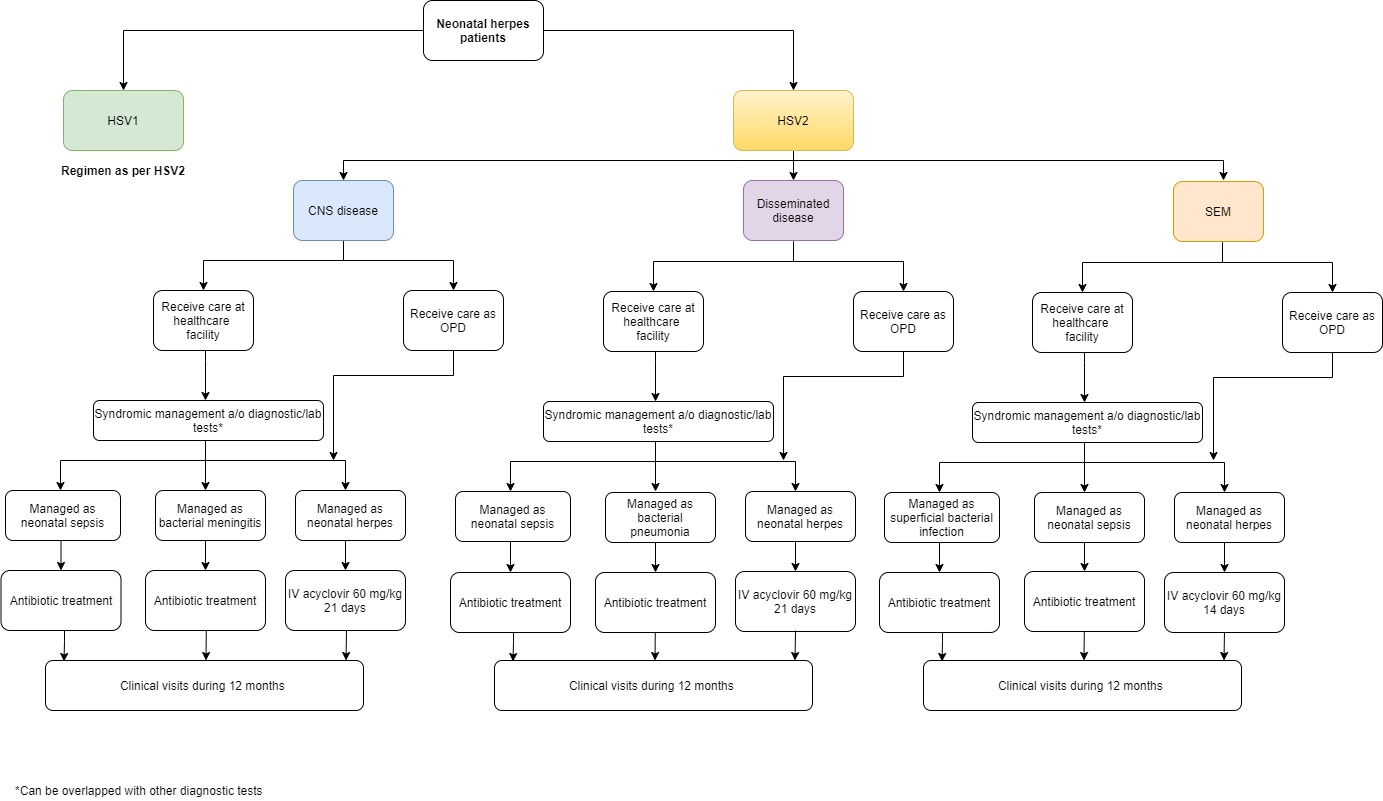
**

**Fig. S3** Overview of healthcare resource utilization related to typical presentations of neonatal herpes.

*Note*: To estimate healthcare resource utilization (HCRU), experts were asked to estimate the proportions of patients that would receive different types of care upon presenting with the typical presentations listed above, regardless of whether these presentations are ever recognized as neonatal herpes. There is also the option that the neonates are not brought for care nor treated.

## **2.2 Neonatal herpes**

All neonatal herpes patients are classified into three major clinical presentations. Approximately one-third of cases present with encephalitis with focal/generalized seizures, bulging fontanel, irritability, lethargy, and poor feeding at 16-19 days of life and categorized as CNS disease. Disseminated disease accounts for about 25% during 10-12 days of life where infants present with respiratory and hepatic failure, often disseminated intravascular coagulation (DIC), and involvement of multiple organs. SEM (skin, eye, and/or mouth) disease entails a vesicular rash at the skin, eye, and/or mouth without CNS or visceral organ involvement, occurring at 10-12 days of life and comprising around 45% of neonatal herpes cases (20% before the introduction of antiviral treatment). Due to overlapping clinical presentations, those with CNS and disseminated diseases may be managed as neonatal sepsis, bacterial meningitis/pneumonia, and/or neonatal herpes, whereas varying proportions of those with SEM disease would be managed as superficial bacterial infection, neonatal sepsis, and/or neonatal herpes. These treatment patterns have been captured in HCRU based on expert interviews. IV acyclovir 60 mg/kg for 21 days is used for those who are managed as neonatal herpes with CNS or disseminated disease while IV acyclovir 60 mg/kg for 14 days is administered for those who are managed as neonatal herpes with SEM disease (Fig. S3 and S4).

HCRU for neonatal herpes was estimated based on expert opinion according to the methodology outlined above. As not all experts were able to provide estimates for neonatal herpes, we used estimates of health care services from LMICs in SEAR for all other LMIC regions apart from the proportion of neonates receiving care and the number of follow-up visits in LMICs in AFR. Given that the proportion of patients receiving care in SEAR LMICs was higher than AFR HIC estimates, estimates from AFR HICs was used for AFR LMICs since it was more relevant.

**
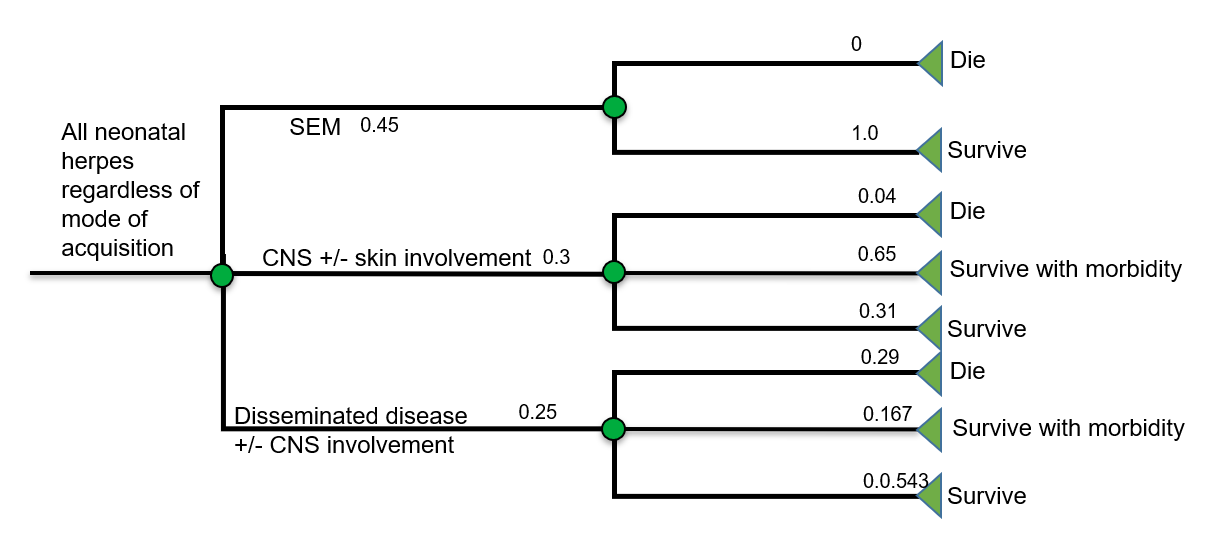
**

**Fig. S4** Overview of neonatal herpes clinical presentations.

**Table S1** Summary of healthcare resource utilization estimates for HSV GUD in adults and adolescents, by region based upon expert opinion.

| **Healthcare resource utilization parameters** | **AFR (HICs)** | **AFR (LMICs)** | **EUR, EMR (HICs)** | **EUR (LMICs)** | **EMR (LMICs)** | **AMR (HICs)** | **AMR (LMICs)** | **WPR, SEAR (HICs)** | **SEAR (LMICs)** | **WPR (LMICs)** |
| --- | --- | --- | --- | --- | --- | --- | --- | --- | --- | --- |
| **1st GUD episode** |  |  |  |  |  |  |  |  |  |  |
| ***Healthcare seeking behavior (%)*** | | | | | | | | | | |
| Proportion seeking care at healthcare facility | 50 | 5 | 50 | 77.5 | 20 | 50 | 50 | 87.5 | 77.5 | 77.5 |
| Proportion seeking care at pharmacy | 5 | 5 | 5 | 5 | 50 | 5 | 5 | 5 | 22.5 | 22.5 |
| *Diagnostic tests (%)* | | | | | | | | | | |
| Clinical/syndromic management of GUD symptoms only | 77.5 | 95 | 50 | 95 | 95 | 5 | 95 | 2 | 95 | 5 |
| HSV viral culture | 1 | NA | 0 | 0 | 5 | 77.5 | NA | 1 | 5 | 5 |
| Polymerase chain reaction (HSV PCR) | 1 | NA | 22.5 | 5 | 5 | 50 | NA | 95 | 5 | 5 |
| HSV serologic test | 22.5 | NA | 0 | 50 | 5 | 50 | NA | 5 | 5 | 77.5 |
| Non-treponemal test(s) for syphilis (RPR, VDRL) | 5 | NA | 5 | 50 | 5 | 95 | 95 | 5 | 50 | 77.5 |
| Rapid treponemal test for syphilis | 5 | NA | 0 | 50 | 5 | 22.5 | 95 | NA | 5 | 77.5 |
| Laboratory-based treponemal test(s) for syphilis | 22.5 | NA | 22.5 | 5 | 5 | 95 | 77.5 | 95 | 5 | 77.5 |
| HIV test | 50 | 95 | 22.5 | 50 | 5 | 95 | 95 | 95 | 95 | 77.5 |
| HSV treatment given/prescribed (%) | 77.5 | 95 | 77.5 | 77.5 | 95 | 77.5 | 50 | 95 | 77.5 | 95 |
| ***Medication regimens (%)*** | | | | | | | | | | |
| Acyclovir 400 mg 3 times daily for 5 days | 0 | 0 | 25 | 0 | 0 | 0 | 0 | 0 | 0 | 0 |
| Acyclovir 400 mg 3 times daily for 7 days | 0 | 100 | 0 | 0 | 25 | 20 | 80 | 0 | 100 | 24 |
| Acyclovir 400 mg 3 times daily for 10 days | 0 | 0 | 25 | 0 | 0 | 0 | 0 | 0 | 0 | 16 |
| Acyclovir 200 mg 5 times daily for 5 days | 0 | 0 | 25 | 0 | 0 | 0 | 0 | 0 | 0 | 0 |
| Acyclovir 200 mg 5 times daily for 7 days | 100 | 0 | 0 | 45 | 25 | 0 | 20 | 0 | 0 | 6 |
| Acyclovir 200 mg 5 times daily for 10 days | 0 | 0 | 25 | 5 | 0 | 0 | 0 | 0 | 0 | 4 |
| Valacyclovir 300 mg 2 times daily for 7 days | 0 | 0 | 0 | 0 | 0 | 0 | 0 | 0 | 0 | 21 |
| Valacyclovir 300 mg 2 times daily for 10 days | 0 | 0 | 0 | 0 | 0 | 0 | 0 | 0 | 0 | 14 |
| Valacyclovir 500 mg 2 times daily for 7 days | 0 | 0 | 0 | 45 | 40 | 10 | 0 | 50 | 0 | 0 |
| Valacyclovir 500 mg 2 times daily for 10 days | 0 | 0 | 0 | 5 | 0 | 0 | 0 | 50 | 0 | 0 |
| Valacyclovir 1 g 2 times daily for 7 days | 0 | 0 | 0 | 0 | 10 | 60 | 0 | 0 | 0 | 0 |
| Valacyclovir 1 g 2 times daily for 10 days | 0 | 0 | 0 | 0 | 0 | 0 | 0 | 0 | 0 | 0 |
| Famciclovir 250 mg 3 times daily for 7 days | 0 | 0 | 0 | 0 | 0 | 10 | 0 | 0 | 0 | 9 |
| Famciclovir 250 mg 3 times daily for 10 days | 0 | 0 | 0 | 0 | 0 | 0 | 0 | 0 | 0 | 6 |
| ***Proportion patients receiving treatment for syphilis (%)*** | 77.5 | 5 | 0 | 0 | 0 | 22.5 | 0 | 5 | 15 | 0 |
| **Recurrent GUD episode** |  |  |  |  |  |  |  |  |  |  |
| ***Healthcare seeking behavior (%)*** | | | | | | | | | | |
| Proportion seeking care at healthcare facility | 22.5 | 22.5 | 22.5 | 50 | 95 | 22.5 | 22.5 | 50 | 50 | 50 |
| Proportion seeking care at pharmacy | 5 | 5 | 5 | 5 | 5 | 5 | 5 | 5 | 22.5 | 22.5 |
| ***Diagnostic tests (%)*** | | | | | | | | | | |
| Clinical/syndromic management of GUD symptoms only | 77.5 | 95 | 100 | 95 | 95 | 22.5 | 95 | 50 | 100 | 77.5 |
| HSV viral culture | 5 | NA | 5 | 0 | 5 | 50 | NA | 5 | 5 | 5 |
| Polymerase chain reaction (HSV PCR) | 5 | NA | 5 | 5 | 5 | 22.5 | NA | 5 | 5 | 5 |
| HSV serologic test | 5 | NA | 0 | 50 | 5 | 22.5 | NA | 5 | 5 | 5 |
| Non-treponemal test(s) for syphilis (RPR, VDRL) | 5 | NA | 5 | 50 | 5 | 5 | 95 | 5 | 22.5 | 5 |
| Rapid treponemal test for syphilis | 5 | NA | 0 | 50 | 5 | 5 | 95 | NA | 5 | 5 |
| Laboratory-based treponemal test(s) for syphilis | 5 | NA | 50 | 5 | 5 | 5 | 77.5 | 65 | 5 | 5 |
| HIV test | 60 | 95 | 50 | 50 | 5 | 22.5 | 95 | 77.5 | 22.5 | 5 |
| HSV treatment given/prescribed | 12.5 | 95 | 22.5 | 77.5 | 95 | 50 | 50 | 22.5 | 50 | 95 |
| HSV medication from home | 5 | 5 | 22.5 | 50 | 95 | 50 | 5 | 22.5 | 50 | 5 |
| ***Medication regimens (%)*** | | | | | | | | | | |
| Acyclovir 400 mg 3 times daily for 3 days | 0 | 0 | 0 | 0 | 0 | 0 | 0 | 0 | 0 | 0 |
| Acyclovir 400 mg 3 times daily for 5 days | 0 | 100 | 80 | 50 | 0 | 0 | 100 | 0 | 0 | 50 |
| Acyclovir 400 mg 3 times daily for 7 days | 90 | 0 | 0 | 0 | 0 | 0 | 0 | 0 | 100 | 0 |
| Acyclovir 800 mg 2 times daily for 3 days | 0 | 0 | 20 | 0 | 0 | 0 | 0 | 0 | 0 | 0 |
| Acyclovir 800 mg 2 times daily for 5 days | 0 | 0 | 0 | 0 | 0 | 40 | 0 | 0 | 0 | 0 |
| Acyclovir 800 mg 2 times daily for 7 days | 0 | 0 | 0 | 0 | 0 | 0 | 0 | 0 | 0 | 0 |
| Valacyclovir 300 mg 2 times daily for 5 days | 0 | 0 | 0 | 0 | 0 | 0 | 0 | 0 | 0 | 24 |
| Valacyclovir 500 mg 2 times daily for 3 days | 0 | 0 | 0 | 0 | 100 | 40 | 0 | 0 | 0 | 0 |
| Valacyclovir 500 mg 2 times daily for 5 days | 0 | 0 | 0 | 50 | 0 | 10 | 0 | 0 | 0 | 11 |
| Valacyclovir 500 mg 2 times daily for 7 days | 10 | 0 | 0 | 0 | 0 | 0 | 0 | 50 | 0 | 0 |
| Valacyclovir 500 mg 2 times daily for 10 days | 0 | 0 | 0 | 0 | 0 | 0 |  | 50 | 0 | 0 |
| Famciclovir 250 mg 3 times daily for 3 days | 0 | 0 | 0 | 0 | 0 | 0 | 0 | 0 | 0 | 0 |
| Famciclovir 250 mg 3 times daily for 5 days | 0 | 0 | 0 | 0 | 0 | 10 | 0 | 0 | 0 | 15 |
| Others | 0 | 0 | 0 | 0 | 0 | 0 | 0 | 0 | 0 | 0 |
| ***Proportion patients receiving treatment for syphilis (%)*** | 50 | 5 | 0 | 0 | 0 | 5 | 0 | 5 | 5 | 0 |
| ***Suppressive therapy*** | | | | | | | | | | |
| Proportion of patients receiving suppressive treatment (%) | 5 | 5 | 5 | 22.5 | 50 | 22.5 | 5 | 22.5 | 5 | 22.5 |
| Average duration of suppressive therapy (years) | 1 | 0.083 | 1 | 0.5 | 0.5 | 1 | 1 | 1 | 0.5 | 0.5 |
| ***Medication regimens (%)*** | | | | | | | | | | |
| Acyclovir 400 mg 2 times daily | 95 | 100 | 90 | 50 | 0 | 20 | 100 | 0 | 100 | 60 |
| Acyclovir 400 mg 3 times daily | 0 | 0 | 6 | 0 | 0 | 0 | 0 | 0 | 0 | 0 |
| Valacyclovir 500 mg once daily | 5 | 0 | 2 | 50 | 90 | 70 | 0 | 98 | 0 | 5 |
| Valacyclovir 1 g once daily | 0 | 0 | 2 | 0 | 10 | 5 | 0 | 2 | 0 | 5 |
| Famciclovir 250 mg 2 times daily | 0 | 0 | 0 | 0 | 0 | 5 | 0 | 0 | 0 | 10 |
| Famciclovir 300 mg once daily | 0 | 0 | 0 | 0 | 0 | 0 | 0 | 0 | 0 | 20 |

AFR, African Region; AMR, Region of the Americas; SEAR, South-East Asian Region; EUR, European Region; EMR, Eastern Mediterranean Region; WPR, Western Pacific Region; HICs, High-Income Countries; LMICs, Low Middle Income Countries; GUD, Genital ulcer disease

**Table S2** Summary of healthcare resource utilization estimates for HSV related to GUD in pregnancy, by region based upon expert opinion.

| **Healthcare resource utilization parameters** | **AFR (HICs)** | **SEAR, AFR (LMICs)** | **EUR, EMR, AMR (HICs)** | **AMR, EMR, EUR (LMICs)** | **WPR, SEAR (HICs)** | **WPR (LMICs)** |
| --- | --- | --- | --- | --- | --- | --- |
| **Genital herpes during pregnancy** | | | | | | |
| Proportion of 1st episode seeking care at healthcare facility (%) | 77.5 | 77.5 | 77.5 | 95 | 95 | 95 |
| Proportion of recurrent episodes seeking care at healthcare facility (%) | 22.5 | 77.5 | 22.5 | 95 | 77.5 | 95 |
| Proportion of recurrences receiving suppressive therapy from 36 weeks of gestation until delivery (%) | 5 | 50 | 5 | 22.5 | 50 | 22.5 |
| Proportion of symptomatic 1st episode receiving suppressive therapy from 36 weeks of gestation until delivery (%) | 22.5 | 77.5 | 95 | 77.5 | 50 | 77.5 |
| Proportion of those with suspected lesions at the time of delivery receiving C-section (%) | 5 | 50 | 5 | 77.5 | 50* | 50 |
| Proportion of symptomatic 1st episode receiving C-section (%) | 5 | 77.5 | 95 | 77.5 | 95* | 77.5 |
| ***Medication regimens (%)*** | | | | | | |
| Acyclovir 400 mg 2 times daily | 100 | 100 | 0 | 0 | 100 |  |
| Acyclovir 400 mg 3 times daily | 0 | 0 | 100 | 100 | 0 | 75 |
| Valacyclovir 300 mg 2 times daily | 0 | 0 | 0 | 0 | 0 | 20 |
| Valacyclovir 500 mg 2 times daily | 0 | 0 | 0 | 0 | 0 | 5 |

*Note:* *SEAR (LMICs) estimate was used for proportion among those with suspected lesions at the time of delivery receiving C-section; EUR (HICs) estimate was used for proportion among those with symptomatic 1^st^ episode receiving C-section

**Table S3** Summary of healthcare resource utilization estimates for HSV related to typical presentations of neonatal herpes, by region based upon expert opinion.

| **HCRU parameters** | **AFR (HICs)** | **AFR (LMICs)** | **EMR, EUR, PAH, SEAR, WPR (HICs)** | **EMR, EUR, AMR, SEAR, WPR (LMICs)** |
| --- | --- | --- | --- | --- |
| **Neonatal herpes** |  |  |  |  |
| **Scenario 1 CNS disease presentations** | | | | |
| Proportion of neonates receiving care at a hospital (%) | 80 | 80 | 100 | 100 |
| Proportion of neonates receiving care at an OPD (%) | 10 | 10 | 0 | 0 |
| Proportion of neonates not receiving care (%) | 10 | 10 | 0 | 0 |
| Proportion of neonates managed as neonatal sepsis (%) | 95 | 77.5 | 95 | 77.5 |
| Proportion of neonates managed as bacterial meningitis (%) | 95 | 77.5 | 95 | 77.5 |
| Proportion of neonates managed as neonatal herpes (%) | 5 | 5 | 5 | 5 |
| ***Scenario 1 and a vesicular rash**** | | | | |
| Proportion of neonates managed as neonatal sepsis (%) | 95 | 50 | 95 | 50 |
| Proportion of neonates managed as bacterial meningitis (%) | 95 | 50 | 95 | 50 |
| Proportion of neonates managed as neonatal herpes (%) | 77.5 | 77.5 | 77.5 | 77.5 |
| ***Diagnostic tests (%)*** | | | | |
| Physical examination | 95 | 100 | 95 | 100 |
| Lumbar puncture | 95 | 50 | 95 | 50 |
| Blood cultures | 95 | 77.5 | 95 | 77.5 |
| CSF cultures | 95 | 50 | 95 | 50 |
| Viral cultures | 50 | 22.5 | 50 | 22.5 |
| HSV PCR of skin lesions | 50 | 5 | 50 | 5 |
| HSV PCR of blood | 50 | 5 | 50 | 5 |
| HSV PCR of CSF | 77.5 | 5 | 77.5 | 5 |
| White blood cell (WBC) count/ complete blood count (CBC) | 95 | 95 | 95 | 95 |
| C-reactive protein (CRP) | 95 | 95 | 95 | 95 |
| ***Medication regimens (%)*** | | | | |
| IV acyclovir 60 mg/kg/day for 14 days | 0 | 0 | 0 | 0 |
| IV acyclovir 60 mg/kg/day for 21 days | 100 | 100 | 100 | 100 |
| Proportion receiving 6-month suppressive HSV antiviral treatment (%) | 0 | 0 | 0 | 0 |
| Average number of follow up visits | 2 | 2 | 6 | 6 |
| **Scenario 2 Disseminated disease presentations** | | | | |
| Proportion of neonates receiving care at a hospital (%) | 80 | 80 | 100 | 100 |
| Proportion of neonates receiving care at an OPD (%) | 10 | 10 | 0 | 0 |
| Proportion of neonates not receiving care (%) | 10 | 10 | 0 | 0 |
| Proportion of neonates managed as neonatal sepsis (%) | 95 | 77.5 | 95 | 77.5 |
| Proportion of neonates managed as bacterial pneumonia (%) | 95 | 77.5 | 95 | 77.5 |
| Proportion of neonates managed as neonatal herpes (%) | 5 | 22.5 | 5 | 22.5 |
| ***Scenario 2 and a vesicular rash**** | | | | |
| Proportion of neonates managed as neonatal sepsis (%) | 95 | 77.5 | 95 | 77.5 |
| Proportion of neonates managed as bacterial pneumonia (%) | 95 | 77.5 | 95 | 77.5 |
| Proportion of neonates managed as neonatal herpes (%) | 77.5 | 77.5 | 77.5 | 77.5 |
| ***Diagnostic tests (%)*** | | | | |
| Physical examination | 95 | 95 | 95 | 95 |
| Lumbar puncture | 95 | 50 | 95 | 50 |
| Blood cultures | 95 | 77.5 | 95 | 77.5 |
| CSF cultures | 95 | 50 | 95 | 50 |
| Viral cultures | 50 | 22.5 | 50 | 22.5 |
| HSV PCR of skin lesions | 50 | 5 | 50 | 5 |
| HSV PCR of blood | 50 | 5 | 50 | 5 |
| HSV PCR of CSF | 50 | 5 | 50 | 5 |
| White blood cell (WBC) count/ complete blood count (CBC) | 95 | 95 | 95 | 95 |
| C-reactive protein (CRP) | 95 | 95 | 95 | 95 |
| ***Medication regimens (%)*** | | | | |
| IV acyclovir 60 mg/kg/day for 14 days | 0 | 0 | 0 | 0 |
| IV acyclovir 60 mg/kg/day for 21 days | 100 | 100 | 100 | 100 |
| Proportion receiving 6-month suppressive HSV antiviral treatment (%) | 0 | 0 | 0 | 0 |
| Average number of follow up visits | 2 | 2 | 6 | 6 |
| **Scenario 3 SEM presentations** | | | | |
| Proportion of neonates receiving care at a hospital (%) | 80 | 80 | 100 | 100 |
| Proportion of neonates receiving care at an OPD (%) | 10 | 10 | 0 | 0 |
| Proportion of neonates not receiving care (%) | 10 | 10 | 0 | 0 |
| Proportion of neonates managed as superficial bacterial infection (%) | 50 | 5 | 50 | 5 |
| Proportion of neonates managed as neonatal sepsis (%) | 95 | 5 | 95 | 5 |
| Proportion of neonates managed as neonatal herpes (%) | 77.5 | 95 | 77.5 | 95 |
| ***Diagnostic tests (%)*** | | | | |
| Physical examination | 95 | 95 | 95 | 95 |
| Lumbar puncture | 77.5 | 5 | 77.5 | 5 |
| Blood cultures | 77.5 | 95 | 77.5 | 95 |
| CSF cultures | 77.5 | 5 | 77.5 | 5 |
| Viral cultures | 50 | 5 | 50 | 5 |
| HSV PCR of skin lesions | 22.5 | 5 | 22.5 | 5 |
| HSV PCR of blood | 22.5 | 5 | 22.5 | 5 |
| HSV PCR of CSF | 50 | 5 | 50 | 5 |
| White blood cell (WBC) count/ complete blood count (CBC) | 77.5 | 95 | 77.5 | 95 |
| C-reactive protein (CRP) | 77.5 | 95 | 77.5 | 95 |
| ***Medication regimens (%)*** | | | | |
| IV acyclovir 60 mg/kg/day for 14 days | 100 | 100 | 100 | 100 |
| IV acyclovir 60 mg/kg/day for 21 days | 0 | 0 | 0 | 0 |
| Proportion receiving 6-month suppressive HSV antiviral treatment (%) | 0 | 0 | 0 | 0 |
| Average number of follow up visits | 1 | 1 | 6 | 6 |

*Note:* SEAR (LMICs) neonatal herpes estimates were used for EMR (LMICs), EUR (LMICs), AMR (LMICs), WPR (LMICs), AFR (LMICs) (except proportion of receiving care in which estimations from AFR (HICs) were applied. Proportion of receiving care from SEAR (LMICs) was higher than AFR (HICs). Therefore, using AFR (HICs) proportion of receiving care estimates for AFR (LMICs) seemed more relevant.

*The scenario with a vesicular rash is applied in the model. The presence of the rash mostly determines the proportion that would initially get treated for neonatal herpes (NH) at all (vs sepsis etc.), but once they are diagnosed or treated for NH under the general scenario, the treatment would be the same

# **Appendix 3: Unit costs**

A unit cost was referred to as a cost per unit of a resource such as a cost per a doctor visit or a monthly wage rate. By considering the societal perspective, unit costs in this study mainly included direct medical costs, direct non-medical costs (i.e. transportation), and indirect costs (i.e. lost wages). Table S4 describes those types of direct and indirect costs based on the framework of the diseases including HSV 2 and HSV1 infection and the population groups of interest comprising adults, people living with HIV, pregnant women, and infants aged 0 to 28 days. Here we explain further detail about the currency conversion, the imputation methods, and the data and their sources.

The original currency of the unit costs was converted to 2016 international dollars (I$) using a published guideline.^12^ The essential steps were to get local currencies, perform inflation adjustment, and then convert to international dollars using the purchasing power parity (PPP). To elaborate, reported local currencies from local countries were inflated/deflated using the local consumer price indices (CPIs) to produce the values in 2016. After that, the values were divided by the PPP of the country to convert to the values in I$ in the year 2016. If the US dollars were reported from non-US countries, the reported USD values were first converted to the values in local currencies by multiplying the value with the exchange rates of the reported year prior to the processes of deflation/inflation and adjusting to I$2016. The exchange rate was based upon the date of 1^st^ July and the year of interest. If original values were reported in I$, the conversion was accomplished in a similar way—changing the I$ values to local currencies by multiplying with the PPP, deflating/inflating to the year 2016 using the local CPIs, and then adjusting to I$ in 2016.

All unit costs in this study were country-specific and they were either obtained from the data sources or imputed. The imputation was needed for only missing data. The imputation methods were done hierarchically as follows. First, the countries with missing data used the average costs of all other countries with the same income level (i.e. low-income, lower-middle income, upper-middle income, or high income), the same region (e.g. Africa), and the same subregion (e.g. western Africa). Second, if the same subregion was not available, the same income level and the same region were used instead. Third, if the same subregion and the same region were not available, the average cost of all other countries with the same income level from the entire world was produced to be the cost of the country lacking data. Fourth, if there was no information at all from any country for a particular income level, we used the ratio methods to construct the costs.

The ratio methods created a proportion of the average cost of a similar product from all countries in a certain income level (e.g., low-income) to the average cost of such product from all countries in the other income level (high-income). Then, such a ratio was used for imputation by multiplying with the known cost. For some cases with richer data, the ratios would be constructed from the same income level and the same region if they were available. Otherwise, the ratio was initiated from a global level (all countries for a particular income level). The following illustrates the ratio methods in an equation.

$$Cost ofX_{i} for {income level}_{j}= \frac{(\sum_{i=1}^{n} Y_{i} for {income level}_{j})/n}{(\sum_{i=1}^{n} Y_{i} for {income level}_{k})/n}* (\sum_{i=1}^{n} X_{i} for {income level}_{k})/n$$

$i=each country$

$$n=number of countries$$

$$j,k=income levels including low, lower-middle, upper-middle, and high$$

From the equation, it reads cost of a product X for a country i from income level j equals the product of the ratio of the average cost of a product Y for income level j to the average cost of a product Y for income level k and the average cost of product X for income level k. For instance, cost of a VDRL-syphilis test for Burundi which is a low-income country (I$5) equals the product of the ratio of the average cost of an HIV diagnosis test from all of low-income countries to the average cost of an HIV diagnosis test from all of upper-middle-income countries (I$10/I$20 =0.5), and the average cost of the VDRL-syphilis test from the upper-middle-income countries (I$ 10).

## **3.1 Unit cost of outpatient visits at a healthcare facility**

Outpatient visits at a healthcare facility for HSV infection were conceptually defined as any visit for HSV-related infection when an individual does not need hospitalization at any kind of a healthcare facility. The unit cost data were taken from “costs per outpatient visit” estimated by WHO-CHOICE (CHOosing Interventions that are Cost Effective).^13^ Briefly, the WHO-CHOICE provided the global cost estimates per outpatient visit using data from the primary and secondary collection using regression models. These outpatient unit costs provide cost estimates related to personnel, capital infrastructure and equipment, standard laboratory-related need, maintenance and other operational costs of the health facility. In the present study, the unit cost of an outpatient visit for each country was the average cost of “the mean value from sample” from all five types of health care facilities including health centres with no beds, health centres with beds, primary hospitals, secondary hospitals, and tertiary hospitals.

## **3.2 Unit cost of outpatient visits at a pharmacy**

The unit cost of a pharmacy visit for a patient was estimated by using pharmacist's salary and assumed pharmacist time. Pharmacist salary reflects healthcare services provided at a pharmacy such as patient evaluation and drug dispensing. It was assumed that a pharmacist spent 5 minutes on average providing care to a patient with HSV infection. Pharmacist salary data were taken from median pharmacist compensation per hour by country from a publicly available website namely [www.salaryexpert.com](http://www.salaryexpert.com).^14^ According to the website, the median pharmacist compensation was the average between the early-career pharmacists (1-3 years of experience) and the senior pharmacists (8 years of experience) from the whole country. Such average was drawn from internal surveys, third-party surveys, and public sources conducted by the salary expert team.

## **3.3 Unit cost of inpatient visits**

The unit cost of an inpatient visit per day was the average estimates from all three types of hospitals including primary hospitals, secondary hospitals, and tertiary hospitals estimated by WHO-CHOICE.^13^ According to WHO-CHOICE, the inpatient cost per day does not include the costs of drugs or diagnostic testing. It includes the hotel components such as personnel, capital infrastructure and equipment, maintenance, general laboratory tests, food, other operational costs of the hospital, which were assumed to be the standard across different diseases and treatments.

## **3.4 Unit costs of laboratory or diagnostic tests**

This study included the following diagnostic or laboratory tests for HSV1 and HSV2 infection: HSV PCR tests, HSV cell culture, HSV serological tests, VDRL tests for syphilis, RPR tests for syphilis, Laboratory-based treponemal tests for syphilis, HIV diagnostic tests, neonatal lumbar puncture, neonatal blood cultures, neonatal CSF cultures neonatal viral cultures, neonatal HSV PCR at skin lesions, blood, and CSF, WBC/CBC, and CRP. Data on laboratory unit costs were searched mainly from PubMed database by using the keywords: laboratory names, cost, and region or income level. In our calculation, the unit costs of the laboratory or diagnostic tests included the staff time and supplies. The average values were used when there were more than one brand or type of laboratory test was found.

## **3.5 Unit cost of counselling**

Unit cost of counselling was estimated from nurses’ salaries and the assumed counselling time of 15 minutes. The nurses’ salaries were calculated from the average index earnings by income levels^15^ and gross domestic products (GDP) per capita in 2016.^16^

## **3.6 Unit costs of treatment and suppressive therapy**

Treatment for HSV infection in this study included only medications. A number of data sources were utilized for searching drug prices. The principal source was from WHO/HAI^17^ and national-agency websites for drug prices listed on the WHO webpage.^18^ The costs of the medications in this study were mainly the generic prices.

## **3.7 Unit cost of transportation**

The prices of the round-trip local tickets for local transportation were used to be the unit costs of transportation. They were obtained from a publicly available website^19^ which was previously used by the WHO team to estimate transportation costs for health care programs.

## **3.8 Wages**

Average wage rates per month of each country were obtained from global wage report 2018/2019 by International Labour Organization.^20^ All costs identified were country-specific nominal wages per month (unadjusted values). The estimates from year 2016 or close to year 2016 were used.

## **3.9 Spending for new cases of HIV/AIDS attributable to HSV infection**

Because HSV-2 can increase risk for HIV infection, the analysis took into account new cases of HIV attributed to HSV-2 in 2016. The unit cost of HIV infection in the present study directly used the published ‘total health spending’ of HIV/AIDS treatment and prevention per capita estimated by the Global Burden of Disease Health Financing Collaborator Network for one year.^21^ In such publication, the cost data of 188 countries were readily available and they were estimated using country-specific data between 1995 to 2015 from the government spending, out-of-pocket, and pre-paid private spending.

## **3.10 Unit costs of disease management on neonates**

There were three types of disease management for neonates with symptoms suspected for neonatal herpes. Those were neonatal sepsis, neonatal bacterial meningitis, and neonatal herpes. The unit costs of neonatal sepsis and neonatal bacterial meningitis included costs related to inpatient stay, intensive care, and medications. The unit cost of neonatal herpes included only inpatient stay and medications. Outpatient costs and laboratory costs were incorporated in the analysis level although they were not combined in the unit cost level. We did not include costs related to health consequences such as neurologic sequelae after having those diseases. A search for studies reporting the unit costs of management of neonates related to neonatal herpes was done in PubMed using the terms of cost and the name of the disease, and/or income levels, and/or regions (e.g. America, West Pacific).

More specifically, the neonatal sepsis treatment in the study reflects the case of early onset neonatal sepsis. The inpatient costs were taken from the WHO-CHOICE. The medication costs were obtained from the same sources used for HSV treatment. The intensive care costs, the inpatient days, the intensive care days, and the antibiotic days were taken from the literature. Missing data were imputed using the same methods considering the same income level having the same estimates. The neonate’s weight was assumed to be 3 kg for all countries. Based on the information from UpToDate and guidelines, the costs of medications were calculated from ampicillin 100mg/kg/dose given every 8 hours and gentamicin 4 mg/kg/dose every 24 hours. Similar to neonatal sepsis, neonatal bacterial meningitis used the same sources to acquire data and assumed 3 kg for neonates when calculating medication doses. The assumed medications included ampicillin 200mg/kg/day, gentamicin 5mg/kg/day and cefotaxime 100mg/kg/day and the assumed antibiotic days were the combined inpatient and intensive care days. There were two treatment options for neonatal herpes depending on the organs infected by HSV. The first treatment scheme was for skin, eye, and mouth: acyclovir 60 mg/kg/day for 14 days of inpatient stay and the second treatment scheme was for CNS and disseminated disease: acyclovir 60 mg/kg/day for 21 days of inpatient stay.

## **3.11 Unit costs of vaginal delivery and caesarean delivery**

Pregnant women who were infected by HSV1 or HSV2 had two options of delivery: vaginal delivery or caesarean delivery. The unit costs of vaginal and caesarean deliveries were referred to the total hospitalization cost during the hospital stay, or the planned costs. The search was done in PubMed using the key words including costs, type of the delivery, and income level (such as low-income countries) or regions (such as Africa). Additional data were obtained from the literature review of HSV infection conducted by the authors of this present study (NC and SL). In the analysis, only the cost difference between vaginal delivery and caesarean delivery was used to evaluate the extra costs incurred by the HSV infection.

**Table S4** Types of unit costs in the analysis.

| **Healthcare-resource/financial parameter** | **Population groups for each country in the main and sensitivity analyses** | | | | | | | | | | |
| --- | --- | --- | --- | --- | --- | --- | --- | --- | --- | --- | --- |
|  | **Main analysis** | | | | | | | | | | |
|  | ***Adults with GUD caused by HSV-2*** | | ***Pregnant women with GUD caused by HSV-2*** | | ***Neonates with herpes infection caused by HSV-2*** | ***Incident HIV cases attributable to HSV-2 infection*** | ***Adults with GUD caused by HSV-1*** | | ***Pregnant women with GUD caused by HSV-1*** | | ***Neonates with herpes infection caused by HSV-1*** |
|  | **First episode** | **Recurrent episode** | **First episode** | **Recurrent episode** |  |  | **First-episode** | **Recurrent episode** | **First-episode** | **Recurrent episode** |  |
| **1. Outpatient visits at a healthcare facility and a pharmacy** | OPD cost | OPD cost | OPD cost | OPD cost | OPD cost | Treatment cost of HIV/AIDS | OPD cost | OPD cost | OPD cost | OPD cost | OPD cost |
| **2. Inpatient visits with hospitalization** | IPD cost | IPD cost | IPD cost  and  Additional cesarean delivery cost | IPD cost  and  Additional cesarean delivery cost | Cost of neonatal sepsis  Cost of neonatal meningitis caused by bacteria  Cost of neonatal herpes |  | IPD cost | IPD cost | IPD cost  and  Additional cesarean delivery cost | IPD cost  and  Additional cesarean delivery cost | Cost of neonatal sepsis  Cost of neonatal meningitis caused by bacteria  Cost of neonatal herpes |
| **3. Laboratory or diagnostic tests** | Laboratory/ diagnostic cost | Laboratory/ diagnostic cost | Laboratory/ diagnostic cost | Laboratory/ diagnostic cost | Laboratory/ diagnostic cost |  | Laboratory/ diagnostic cost | Laboratory/ diagnostic cost | Laboratory/ diagnostic cost | Laboratory/ diagnostic cost | Laboratory/ diagnostic cost |
| **4. Counseling** | Counseling cost | Counseling cost | Counseling cost | Counseling cost | Counseling cost for parents |  | Counseling cost | Counseling cost | Counseling cost | Counseling cost | Counseling cost for parents |
| **5. Treatment** | Medication cost | Medication cost | Medication cost | Medication cost | Medication cost |  | Medication cost | Medication cost | Medication cost | Medication cost | Medication cost |
| **6. Suppressive therapy** | Medication cost | Medication cost | Medication cost | Medication cost | Medication cost |  | Medication cost | Medication cost | Medication cost | Medication cost | Medication cost |
| **7. Transportation for outpatient and inpatient visits** | Transportation cost | Transportation cost | Transportation cost | Transportation cost | Transportation cost |  | Transportation cost | Transportation cost | Transportation cost | Transportation cost | Transportation cost |
| **8. Time lost from work** | Wage rate | Wage rate | Wage rate | Wage rate | Wage rate of parents |  | Wage rate | Wage rate | Wage rate | Wage rate | Wage rate of parents |

# **Appendix 4: Healthcare spending attributable to disease**

To estimate attributable spending for HSV in pregnancy and HIV, we multiplied the healthcare spending attributable to ill health for each health condition by the population attributable fraction for each risk factor and that health condition. This was performed for each health condition under investigation, and for each of the age stratum and sex groups

## **4.1 Comparison of estimates of economic burden associated with HSV with existing literature**

Comparison on estimates of economic burden associated with HSV with existing literature and estimates from the present study is provided in Table S5. In accordance with the Silva study^24^, which estimated the economic losses due to genital herpes in 90 LMICs, we derived the economic burden for the same 90 countries in our study. We were able to perform an economic burden estimation based on 90 countries exactly matched to the countries reported in Silva study. We calculated economic burden including direct medical, direct non-medical, and indirect costs of GUD and HIV due to HSV-2. The total burden attributable to HSV-2 within these countries in our study amounts to I$20 billion. This is lower than the findings of Silva et al. who reported $29 billion in losses, including absenteeism, as highlighted in their abstract.

**Table S5** Estimates of economic burden associated with HSV-2 calculated by matching 90 low- and middle-income countries reported in existing literature.

| **WHO regions** | **No. of countries** | **Direct medical+Direct non-medical+Indirect cost** | | | **Direct medical cost** | | |
| --- | --- | --- | --- | --- | --- | --- | --- |
|  |  | **GUD** | **HIV** | **All** | **GUD** | **HIV** | **All** |
| Globally | 90 | 19,340 | 263 | 19,603 | 10,831 | 207 | 11,039 |
| Africa | 36 | 3,254 | 219 | 3,473 | 1,627 | 169 | 1,796 |
| Americas | 17 | 2,223 | 24 | 2,248 | 1,547 | 22 | 1,570 |
| Eastern Mediterranean | 12 | 1,473 | 1 | 1,474 | 657 | 0 | 658 |
| Europe | 10 | 1,435 | 7 | 1,442 | 867 | 6 | 873 |
| South-East Asia | 8 | 3,632 | 6 | 3,638 | 2,193 | 4 | 2,198 |
| Western Pacific | 7 | 7,322 | 6 | 7,328 | 3,939 | 5 | 3,944 |

*Note:* All cost presented in International Dollar (I$) (millions)

**Table S6** Breakdown of cost associated with HSV-2.

| **Global and WHO regions** | **Total economic burden** | **OPD visits** | **IPD visits** | **Laboratory tests** | **Counselling** | **Treatment** | **Suppressive therapy** | **Transportation** | **Lost productivity** | **New HIV** | **Neonatal** |
| --- | --- | --- | --- | --- | --- | --- | --- | --- | --- | --- | --- |
| **Globally** | 31,213 | 5,734 | 2,594 | 5,064 | 1,107 | 332 | 3,871 | 972 | 11,134 | 352 | 53 |
| **Africa** | 3,580 | 540 | 3 | 940 | 143 | 23 | 32 | 200 | 1,474 | 219 | 6 |
| **Americas** | 6,586 | 1,171 | 1,135 | 1,293 | 165 | 63 | 720 | 135 | 1,803 | 78 | 22 |
| **Eastern Mediterranean** | 1,754 | 338 | 78 | 93 | 90 | 9 | 253 | 72 | 815 | 0.8 | 3 |
| **Europe** | 4,091 | 854 | 568 | 560 | 127 | 10 | 537 | 93 | 1,292 | 41 | 10 |
| **South-East Asia** | 3,886 | 770 | 152 | 994 | 228 | 24 | 207 | 255 | 1,250 | 6 | 1.1 |
| **Western Pacific** | 11,317 | 2,062 | 657 | 1,185 | 353 | 204 | 2,121 | 217 | 4,500 | 8 | 10 |

*Note:* All cost presented in International Dollar (I$) (millions)

**Table S7** Breakdown of cost associated with HSV-1.

| **WHO regions** | **Total economic burden** | **OPD visits** | **IPD visits** | **Laboratory tests** | **Counselling** | **Treatment** | **Suppressive therapy** | **Transportation** | **Lost productivity** | **Neonatal** |
| --- | --- | --- | --- | --- | --- | --- | --- | --- | --- | --- |
| **Globally** | 4,048 | 196 | 2,095 | 218 | 17 | 123 | 222 | 22 | 1,095 | 61 |
| **Africa** | 0.0 | - | - | - | - | - | - | - | - | 0.0 |
| **Americas** | 1,991 | 84 | 1,170 | 131 | 6 | 78 | 68 | 9 | 410 | 36 |
| **Eastern Mediterranean** | 196 | 17 | 35 | 5 | 4 | 13 | 45 | 4 | 72 | 1.5 |
| **Europe** | 915 | 66 | 459 | 40 | 7 | 11 | 84 | 7 | 229 | 13 |
| **South-East Asia** | 16 | 0.6 | 7 | 1.0 | - | 0.1 | 0.1 | 0.2 | 7 | 0.3 |
| **Western Pacific** | 931 | 29 | 425 | 41 | 0.4 | 21 | 25 | 2 | 377 | 11 |

*Note:* All cost presented in International Dollar (I$) (millions)

**Table S8** Annual burden of cost related to HSV in 2016.

| **Global and WHO regions** | **HSV-2** | | | **HSV-1** | | |
| --- | --- | --- | --- | --- | --- | --- |
|  | **Direct medical** | **Direct non-medical** | **Indirect** | **Direct medical** | **Direct non-medical** | **Indirect** |
| **Genital ulcer disease** |  |  |  |  |  |  |
| **Globally** | 19,038 | 977 | 11,198 | 2,924 | 22 | 1,095 |
| **Africa** | 1,853 | 204 | 1,522 | 0.02 | 0.00 | 0.01 |
| **Americas** | 4,641 | 135 | 1,809 | 1,569 | 9 | 413 |
| **Eastern Mediterranean** | 866 | 72 | 816 | 120 | 4 | 72 |
| **Europe** | 2,702 | 93 | 1,296 | 678 | 7 | 231 |
| **South-East Asia** | 2,380 | 255 | 1,251 | 9 | 0.2 | 7 |
| **Western Pacific** | 6,596 | 217 | 4,503 | 549 | 2 | 380 |
|  |  |  |  |  |  |  |
| **Neonatal herpes** |  |  |  |  |  |  |
| **Globally** | 44 | 0.04 | 9 | 52 | 0.03 | 8 |
| **Africa** | 4 | 0.01 | 3 | 0.02 | 0.00 | 0.01 |
| **Americas** | 20 | 0.01 | 2 | 32 | 0.02 | 4 |
| **Eastern Mediterranean** | 3 | 0.01 | 0.9 | 1.1 | 0.00 | 0.4 |
| **Europe** | 9 | 0.00 | 1.1 | 11 | 0.00 | 1.4 |
| **South-East Asia** | 0.8 | 0.00 | 0.3 | 0.2 | 0.00 | 0.0 |
| **Western Pacific** | 8 | 0.01 | 2 | 8 | 0.01 | 2 |
|  |  |  |  |  |  |  |
| **HIV attributable to HSV** |  |  |  |  |  |  |
| **Globally** | 292 | 5 | 55 | - | - | - |
| **Africa** | 169 | 4 | 46 | - | - | - |
| **Americas** | 74 | 0.3 | 4 | - | - | - |
| **Eastern Mediterranean** | 0.6 | 0.01 | 0.1 | - | - | - |
| **Europe** | 37 | 0.2 | 3 | - | - | - |
| **South-East Asia** | 5 | 0.1 | 0.9 | - | - | - |
| **Western Pacific** | 7 | 0.0 | 1.2 | - | - | - |

*Note:* All cost presented in International Dollar (I$) (millions)

**Table S9** Distributional economic impact of HSV from probabilistic analyses**.**

| **WHO regions** | **Genital ulcer disease due to HSV-1** | **Genital ulcer disease due to HSV-2** | **Neonatal herpes due to HSV-1** | **Neonatal herpes due to HSV-2** | **HIV-attributable to HSV** |
| --- | --- | --- | --- | --- | --- |
| **Globally** | 4,086 (2,262-7,285) | 31,133 (20,091-42,774) | 64 (30-110) | 55 (31-98) | 352 (193-539) |
| **Africa** | - | 3,374 (1,975-5,078) | 0.03 (0.01-0.05) | 7 (3-11) | 219 (50-406) |
| **Americas** | 1,970 (846-4,593) | 6,516 (3,215-11,896) | 38 (10-86) | 22 (6-58) | 78 (27-124) |
| **Eastern Mediterranean** | 199 (91-343) | 1,773 (1,223-2,366) | 2 (0.5-3) | 3 (2-6) | 0.8 (0.4-1.3) |
| **Europe** | 916 (540-1,477) | 4,172 (2,704-6,276) | 13 (8-22) | 10 (6-16) | 40 (23-57) |
| **South-East Asia** | 17 (2-54) | 3,997 (594-9,590) | 0.3 (0.03-0.9) | 1.5 (0.2-4) | 6 (0.5-14) |
| **Western Pacific** | 984 (132-3,435) | 11,300 (4,540-21,486) | 11 (2-27) | 10 (3-21) | 8 (2-15) |

*Note:* All cost presented in International Dollar (I$) (millions) and 95% CI

**Table S10** Distributional economic impact (in millions I$) of HSV assuming idealistic practice where treatment guidelines were adhered to.

| **WHO regions** | **Total economic burden** | **OPD visits** | **IPD visits** | **Laboratory tests** | **Counselling** | **Treatment** | **Suppressive therapy** | **Transportation** | **Lost productivity** |
| --- | --- | --- | --- | --- | --- | --- | --- | --- | --- |
| **Globally** | 79,877 | 14,866 | 4,690 | 14,923 | 2,869 | 105 | 11,710 | 1,930 | 28,785 |
| **Africa** | 13,265 | 1,595 | 3 | 3,600 | 547 | 13 | 2,558 | 512 | 4,437 |
| **Americas** | 24,471 | 5,185 | 2,306 | 5,871 | 751 | 60 | 1,849 | 502 | 7,948 |
| **Eastern Mediterranean** | 3,982 | 606 | 113 | 216 | 147 | 8 | 1,340 | 93 | 1,459 |
| **Europe** | 12,193 | 2,627 | 1,027 | 1,603 | 360 | 14 | 2,296 | 235 | 4,032 |
| **South-East Asia** | 5,495 | 1,009 | 159 | 1,241 | 358 | 1 | 609 | 264 | 1,854 |
| **Western Pacific** | 20,472 | 3,844 | 1,081 | 2,393 | 706 | 10 | 3,058 | 324 | 9,056 |

# **References**

1. Looker KJ, Johnston C, Welton NJ, et al. The global and regional burden of genital ulcer disease due to herpes simplex virus: a natural history modelling study. *BMJ Global Health* 2020;5(3):e001875. doi: 10.1136/bmjgh-2019-001875

2. James SL, Abate D, Abate KH, et al. Global, regional, and national incidence, prevalence, and years lived with disability for 354 diseases and injuries for 195 countries and territories, 1990–2017: a systematic analysis for the Global Burden of Disease Study 2017. *The Lancet* 2018;392(10159):1789-858. doi: 10.1016/S0140-6736(18)32279-7

3. Looker KJ, Welton NJ, Sabin KM, et al. Global and regional estimates of the contribution of herpes simplex virus type 2 infection to HIV incidence: a population attributable fraction analysis using published epidemiological data. *The Lancet Infectious Diseases* 2020;20(2):240-49. doi: 10.1016/S1473-3099(19)30470-0

4. UN AIDS. Key population atlas.

5. United Nations Department of Economic and Social Affairs. Population Division, Population Estimates and Projections Section.

6. Betran AP, Ye J, Moller A-B, et al. Trends and projections of caesarean section rates: global and regional estimates. *BMJ Global Health* 2021;6(6):e005671. doi: 10.1136/bmjgh-2021-005671

7. Stankiewicz Karita HC, Moss NJ, Laschansky E, et al. Invasive Obstetric Procedures and Cesarean Sections in Women With Known Herpes Simplex Virus Status During Pregnancy. *Open forum infectious diseases* 2017;4(4):ofx248-ofx48. doi: 10.1093/ofid/ofx248

8. Looker KJ, Magaret AS, May MT, et al. First estimates of the global and regional incidence of neonatal herpes infection. *The Lancet Global Health* 2017;5(3):e300-e09. doi: 10.1016/S2214-109X(16)30362-X

9. Sheffield JS, Hill JB, Hollier LM, et al. Valacyclovir prophylaxis to prevent recurrent herpes at delivery: a randomized clinical trial. *Obstet Gynecol* 2006;108(1):141-147. doi:10.1097/01.AOG.0000219749.96274.15

10. Watts DH, Brown ZA, Money D, et al. A double-blind, randomized, placebo-controlled trial of acyclovir in late pregnancy for the reduction of herpes simplex virus shedding and cesarean delivery. *Am J Obstet Gynecol* 2003;188(3):836-843. doi:10.1067/mob.2003.185

11. Brown ZA, Selke S, Zeh J, et al. The acquisition of herpes simplex virus during pregnancy. *N Engl J Med* 1997;337(8):509-515. doi:10.1056/NEJM199708213370801

12. Turner HC, Lauer JA, Tran BX, et al. Adjusting for Inflation and Currency Changes Within Health Economic Studies. *Value Health* 2019;22(9):1026-1032. doi:10.1016/j.jval.2019.03.021

13. Stenberg K, Lauer JA, Gkountouras G, et al. Econometric estimation of WHO-CHOICE country-specific costs for inpatient and outpatient health service delivery. *Cost Eff Resour Alloc* 2018;16:11. doi:10.1186/s12962-018-0095-x

14. Expert S. Pharmacist salaries by country 2021 [Available from: https://www.salaryexpert.com/salary/browse/countries/pharmacist accessed February 26, 2021.

15. Serje J, Bertram MY, Brindley C, et al. Global health worker salary estimates: an econometric analysis of global earnings data. *Cost Eff Resour Alloc* 2018;16:10. doi:10.1186/s12962-018-0093-z

16. Bank W. GDP per capita 2020 [Available from: Worldbank https://data.worldbank.org/indicator/NY.GDP.PCAP.PP.CD accessed April 8th, 2020.

17. WHO/HAI. Database of medicine prices, availability, affordability and price components 2008 [Available from: https://www.haiweb.org/MedPriceDatabase/ accessed April 5th, 2020.

18. World Health Organization. Medicine price information sources 2021 [Available from: https://www.who.int/teams/health-product-and-policy-standards/medicines-selection-ip-and-affordability/affordability-pricing/med-price-info-source accessed April 20th, 2021.

19. Numbeo. Prices by country with one-way ticket (local transport) 2021 [Available from: https://www.numbeo.com/cost-of-living/prices_by_country.jsp?displayCurrency=USD&itemId=24&itemId=20&itemId=18&itemId=109&itemId=108&itemId=107&itemId=206&itemId=25 accessed February 18th, 2021.

20. Organization IL. Global Wage Report 2018/19 2018 [Available from: https://www.ilo.org/global/research/global-reports/global-wage-report/WCMS_650568/lang--en/index.htm accessed February 25th, 2020.

21. Candel FJ, Borges Sá M, Belda S, et al. Current aspects in sepsis approach. Turning things around. *Rev Esp Quimioter* 2018;31(4):298-315.

22. Szucs TD, Berger K, Fisman DN, et al. The estimated economic burden of genital herpes in the United States. An analysis using two costing approaches. *BMC Infectious Diseases* 2001;1(1):5. doi: 10.1186/1471-2334-1-5

23. Fisman DN, Lipsitch M, Hook EW, 3rd, et al. Projection of the future dimensions and costs of the genital herpes simplex type 2 epidemic in the United States. *Sex Transm Dis* 2002;29(10):608-622. doi:10.1097/00007435-200210000-00008

24. Silva S, Ayoub HH, Johnston C, et al. Estimated economic burden of genital herpes and HIV attributable to herpes simplex virus type 2 infections in 90 low- and middle-income countries: A modeling study. *PLoS Med* 2022;19(12):e1003938. doi:10.1371/journal.pmed.1003938
